# Three New *Orbivirus* Species Isolated from Farmed White-Tailed Deer (*Odocoileus virginianus*) in the United States

**DOI:** 10.3390/v12010013

**Published:** 2019-12-20

**Authors:** Mohammad Shamim Ahasan, Kuttichantran Subramaniam, Juan M. Campos Krauer, Katherine A. Sayler, Julia C. Loeb, Olivia F. Goodfriend, Hannah M. Barber, Caroline J. Stephenson, Vsevolod L. Popov, Remi N. Charrel, Samantha M. Wisely, Thomas B. Waltzek, John A. Lednicky

**Affiliations:** 1 Department of Infectious Diseases and Immunology, College of Veterinary Medicine, University of Florida, Gainesville, FL 32610, USA; shamim@hstu.ac.bd (M.S.A.); kuttichantran@ufl.edu (K.S.); tbwaltzek@ufl.edu (T.B.W.); 2 Emerging Pathogens Institute, University of Florida, Gainesville, FL 32610, USA; jloeb@phhp.ufl.edu (J.C.L.); c.stephenson@ufl.edu (C.J.S.); remi.charrel@univ-amu.fr (R.N.C.); wisely@ufl.edu (S.M.W.); 3 Faculty of Veterinary and Animal Sciences, Hajee Mohammad Danesh Science and Technology University, Dinajpur 5200, Rangpur, Bangladesh; 4 Department of Wildlife Ecology and Conservation, University of Florida, Gainesville, FL 32611-0430, USA; jmcampos@ufl.edu (J.M.C.K.); ksayler1@gmail.com (K.A.S.); ogoodfriend@ufl.edu (O.F.G.); 5 Department of Large Animal Clinical Sciences, College of Veterinary Medicine, University of Florida, Gainesville, FL 32608, USA; barberh@ufl.edu; 6 Department of Environmental and Global Health, College of Public Health and Health Professions, University of Florida, Gainesville, FL 32610-0188, USA; 7 Center for Biodefense and Emerging Infectious Diseases, Institute for Human Infections and Immunity, The University of Texas Medical Branch, Galveston, TX 77555-0609, USA; vpopov@UTMB.EDU; 8 Unité des Virus Emergents (UVE: Aix-Marseille Univ, IRD 190, INSERM U1207, IHU Méditerranée Infection), Aix Marseille Universite, 13000 Marseille, France

**Keywords:** deer farming, *Odocoileus virginianus*, orbivirus, reovirus, white-tailed deer

## Abstract

We report the detection and gene coding sequences of three novel *Orbivirus* species found in six dead farmed white-tailed deer in the United States. Phylogenetic analyses indicate that the new orbiviruses are genetically closely related to the Guangxi, Mobuck, Peruvian horse sickness, and Yunnan orbiviruses, which are thought to be solely borne by mosquitos. However, four of the six viruses analyzed in this work were found as co-infecting agents along with a known cervid pathogen, epizootic hemorrhagic disease virus-2 (EHDV-2), raising questions as to whether the new viruses are primary pathogens or secondary pathogens that exacerbate EHDV-2 infections. Moreover, EHDV-2 is known to be a *Culicoides*-borne virus, raising additional questions as to whether *Culicoides* species can also serve as vectors for the novel orbiviruses, if mosquitoes can vector EHDV-2, or whether the deer were infected through separate bites by the insects. Our findings expand knowledge of the possible viral pathogens of deer in the United States. Moreover, due to the close genetic relatedness of the three new orbiviruses to viruses that are primary pathogens of cattle and horses, our findings also underscore a crucial need for additional research on the potential role of the three new orbiviruses as pathogens of other animals.

## 1. Introduction

The cervid farming industry is a fast growing industry in rural Florida and other parts of North America [1]. This venture is significant in scope because the industry generated almost US$8 billion for the U.S. economy and employed > 56,000 people by the year 2017 [1]. In the United States, cervid farming includes reindeer (*Rangifer tarandus*) in Alaska, elk (*Cervus elaphus*) in the Midwest, and white-tailed deer (*Odocoileus virginianus*) in many states such as Florida, Pennsylvania, and Texas. White-tailed deer are the most commonly farmed deer species in the continental United States [2]. Deer farming has positive impacts on local economies, and game preserves have the potential to conserve wild lands that might otherwise be developed commercially; however diseases can be passed between wild and farmed populations with potential impacts to both populations. As in other livestock systems, the spread of infectious diseases among wild and farmed deer can occur through arthropod vector-borne transmission routes, via inhalation of aerosolized virus particles, through nose-nose contact between farmed and wild deer, or through contact with fomites that can be wind-borne or spread through naturally occurring disasters. Appropriate biosecurity measures can reduce the risk of transmission of many pathogens, and therefore reduce the economic and biological impacts of disease outbreaks. These epizootic events can impact local economies as in the case of chronic wasting disease, and spillover transmission can impact wildlife communities [3,4].

Bluetongue virus (BTV) and epizootic hemorrhagic disease virus (EHDV) are orbiviruses that are transmitted by *Culicoides* spp., and these viruses can cause high morbidity and mortality in farmed and wild populations of white-tailed deer in North America [4,5]. Of note, both viruses also affect other wildlife species including mule deer (*Odocoileus hemionus*), pronghorn (*Antilocapra americana*), elk, mountain goat (*Oreamnos americanus*), and bighorn sheep (*Ovis canadensis*) [6], as well as domestic livestock such as cattle [7]. Both viruses circulate in Florida where they are considered endemic in wild populations [5,8,9,10,11], yet cause considerable production losses in farmed populations. Another reovirus, i.e., mammalian orthoreovirus type 2, has recently been reported to be associated with mortality of farmed white-tailed deer fawn in Florida, likely exemplifying yet another important reovirus pathogen of farmed white-tailed deer populations [12].

Despite the economic hardship these reoviruses impose on Florida farmed deer, the viral diseases of farmed deer in Florida and elsewhere are underexplored, and thus a better understanding of (1) the potential infectious diseases that may arise in deer farms, and (2) effective methods to curtail or prevent these diseases, are essential for economic profitability and to minimize the risk of spreading infectious diseases to susceptible wildlife and farmed animals [4]. Identification of the reoviruses in circulation among cervids is essential for the production of preventive vaccines, and for those that are arthropod-borne, control of their vectors.

Viruses of the family *Reoviridae* (“reoviruses”) are non-enveloped, icosahedral viruses that have a triple-capsid structure, and may appear spherical in shape. There are 15 genera of reoviruses, and these are divided into two subfamilies: *Spinareovirinae,* which have relatively large spikes or turrets situated at the 12 icosahedral vertices of either the virion or core particle, and *Sedoreovirinae*, which do not have large surface projections on their virions or core particles, giving them an almost spherical or “smooth” appearance. The genus *Orbivirus* is 1 of the 15 genera within the family *Reoviridae,* and belongs in the subfamily *Sedoreovirinae*. Currently, there are 22 recognized *Orbivirus* species (representing 22 distinct virus serogroups) that have been recognized by the International Committee for the Taxonomy of Viruses (ICTV), and at least 10 unclassified or unassigned viruses [13].

Orbivirus genomes consist of 10 to 12 dsRNA segments that encode seven major structural proteins (virion proteins, VP1–VP7) and 3 to 5 major non-structural proteins (NS1-NS5); one copy of each segment is packaged per virion [13,14,15]. The virus genomic RNA (vgRNA) segments are mostly monocistronic; a few have two or three in-frame initiation codons that lead to expression of additional open-reading frames (ORFs), apparently through leaky scanning [13]. In addition, at least for BTV, a frame-shift initiation codon in one of the genomic segments leads to production of NS4 and Vp6 (helicase). The 10 to 12 genomic segments (Seg) occur in three distinct size classes based on their molecular weight (MW), and these are readily identified through their electrophoretic mobility (i.e., by their electropherogram patterns). These segments are classified as large (L1, L2, and L3), medium (M1, M2, and M3), and small (S1, S2, S3 and S4) and in order of decreasing MW as Seg-1 to Seg-10. The orbivirus *RNA-dependent RNA polymerase* (*RdRp*) gene is encoded by Seg-1, the protein is traditionally termed virus protein 1 (VP1), and its gene traditionally referred to as the *VP1* instead of *RdRp* gene. The amino acid (aa) sequence of VP1 (RdRp protein) is an important marker for orbivirus classification. An aa identity > 30% with that of other characterized orbiviruses is required to be a member of the genus *Orbivirus*. Available data suggests that isolates from different genera usually have < 26% aa identity in comparisons between their RdRps, while within a single genus identities are usually > 33% [13,14]. Of note, the terminology for orbivirus genome segment numeration and protein designation has not been uniform; some laboratories classify orbivirus proteins according to the size of the proteins, whereas others use the size of genome segments from which they are encoded [16]. In this manuscript, for each vgRNA electropherogram position, the protein encoded has been identified in silico, and thus correlates with the size of its coding segment. The aa sequence of the sub-core protein (T2 protein, traditionally referred to as VP3) is encoded by Seg-2, and is used to classify species within genera. Orbiviruses within a single species group show greater than 91% identity of their T2 aa sequences [13]. Seg-3 encodes the major outer capsid protein (VP2), and viruses within the same species have > 76% nucleotide identity while those in different species usually have < 74% identity, and these differences are also reflected in the amino acid sequences of the viral proteins [13]. Of note, VP2 is the major determinant of serotype, with a minor role for VP5 [13,17,18].

Orbiviruses occur globally and infect a wide variety of vertebrate hosts, including bats, birds, marsupials, rodents, sloths, human and non-human primates, and wild and domestic ruminants and equids. They are common in insects and ticks, where their pathogen status is under-explored and thus not clear, but select viruses can cause diseases in vertebrates including humans. Unlike the reoviruses that replicate only in vertebrate species and are transmitted between hosts by respiratory or fecal–oral routes, orbiviruses replicate in both hematophagous vectors (*Culicoides* biting midges, anopheline or culicine mosquitoes, phlebotomine sandflies, and ticks, depending on the virus) and vertebrate hosts. They replicate in and are transmitted between their vertebrate and arthropod vectors. Some orbiviruses such as African horse sickness virus, BTV, EHDV, and Peruvian horse sickness virus (PHSV) are considered serious animal pathogens, while a few such as the Kemerovo, Lebombo, Orungo, and Tribeč viruses are considered “moderate” human pathogens [19].

The University of Florida (UF) Cervidae Health Research Initiative (CHeRI) is a multidisciplinary entity focused on improving the health and production of captive and farmed cervids of Florida. One research focus is on the identification of viruses that impact the health of Florida’s farmed deer. Florida deer farmers can access free diagnostic assays by contacting CHeRI when they have a deceased deer on their farm. Deer farmers have two options, to collect and send samples to the CHeRI lab or ask a technician to perform a field necropsy of the animal. Upon request, the CHeRI also assists with the identification of deer viruses affecting farmed deer outside of Florida.

We report the detection and characterization of three novel orbivirus species from farmed white-tailed deer that were found dead in their pens. To our knowledge, none of these viruses have previously been reported in North America.

## 2. Materials and Methods

### 2.1. Field Collection of Deer Organ and Tissue Specimens

CHeRI provides guidelines on its website for the collection of organs or tissue specimens from dead deer and their shipment to UF for diagnostic pathology work: http://www.wec.ufl.edu/CHeRI/diagnostics/. A non-technical movie is also available: https://www.youtube.com/watch?v=83UKkeXYWik&feature=youtu.be. As possible (depending on specimen integrity and type, and storage procedures), histopathology, microbiology, and virology analyses are performed. Briefly, during field necropsy and gross examination, diseased portions of organs (typically heart, kidney, liver, lungs, and the spleen) are collected as soon as possible after an animal’s death and placed into sterile polyethylene Whirl-Pak^TM^ bags (Nasco, Ft. Atkinson, WI, USA). When possible, whole peripheral blood is collected from recently deceased animals and injected into EDTA blood collection tubes (K_2_EDTA tubes, Becton, Dickinson, and Company, Franklin Lakes, New Jersey, USA). Blood is used for initial RT-PCR screens for the presence of BTV and EHDV vgRNAs, as: (1) BTV and EHDV bind to and remain associated with red blood cells, and (2) RNA purification from blood is relatively straightforward using a variety of commercial RNA purification kits, and thus RT-PCR tests for BTV and EHDV vgRNAs can be promptly initiated from blood specimens for rapid reporting of results to deer farmers. Because of post-mortem peripheral blood coagulation, cardiac blood is collected instead and analyzed for blood-borne pathogens from animals dead for more than two hours. In that instance, an aliquot of cardiac blood is obtained by syringe then injected into EDTA blood collection tubes. After collection, anticoagulated blood (in EDTA blood collection tubes) is maintained refrigerated including during shipment to CHeRI for virology tests. If the RT-PCR tests are negative for BTV and EHDV vgRNAs, the tests are repeated using RNA extracted from spleen tissue. Tissue and organ samples recovered from newly deceased animals are kept refrigerated for histopathology analyses, and microbiology (bacteriology and mycology) and virology work-up. If decomposition of tissues is evident, tissue and organ specimens are transported on cool packs to the UF for microbiology (bacteriology and mycology) and virology work-up only. For virus isolation, emphasis to date at the CHeRI-affiliated laboratories has been on the detection and isolation of BTV and EHDV, as to date, they have been the most common agents of virus-induced mortality in the farmed deer of Florida. Based on our cumulative experiences, both EHDV and BTV can be readily isolated from spleen tissues collected within 48 h of death of cervids that have died of hemorrhagic disease; we have not attempted to isolate the viruses from spleen tissues collected at later time points. All work was approved by the Institutional Animal Care and Use Committee at the University of Florida (IACUC Protocol Numbers 201609390, initiated 24 May 2016, and 201909390, initiated 21 March 2019).

### 2.2. White-Tailed Deer History and Specimens Collected for Tests

Background information regarding each animal is provided below; the prefix “OV” for each animal identification number denotes *Odocoileus virginianus*.

**Figure 1:**
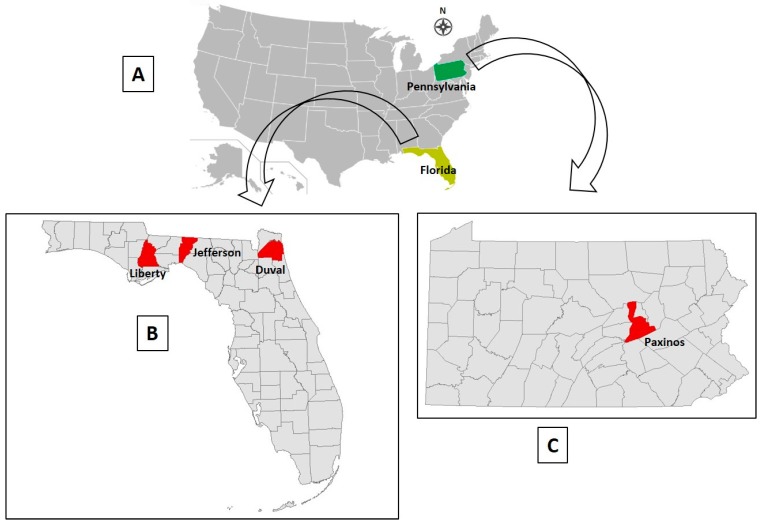
Geographic information regarding white-tailed deer infection sites. (**A**) Map location of the two states (Florida and Pennsylvania) wherein the dead deer of this study originated. (**B**) Location of counties in Florida that contained the deer farms of this report. (**C**) Location of county in Pennsylvania that contained a deer farm in this report.

**OV610.** On August 30, 2017, a farmed 2-year old buck in Paxinos County, Pennsylvania (Figure 1), was found dead in its pen. The farm’s owner noted it was healthy, appearing normal the previous day. A field necropsy was performed by a veterinarian on August 31, 2017, whereupon heart, intestine, liver, lung, kidney, skeletal muscle, spleen, ocular fluid, and rumen contents were collected and sent to the CHeRI for possible diagnostic microbiology (culture), histopathology, and virology evaluations. The spleen was used for virus isolation attempts, as either BTV or EHDV was suspected to be the causative agent, and as noted in Section 2.1 above, either virus can be isolated from a spleen collected < 48 h post-mortem.

**OV617.** On September 10, 2017, a 3-month old fawn (sex not specified by farmer) in Jefferson County, northwest Florida (Figure 1), died. The animal was observed to be ill the previous day, when it was administered a dose of Draxxin (tulathromycin), a triamilide antibiotic indicated for the treatment of bovine and swine bacterial respiratory diseases and bovine foot-rot. Draxxin is also used in deer farming to help control bacterial infections of the lung, as pneumonia is a common consequence of EHDV infection. On September 11, postmortem heart, liver, lung, kidney, and spleen tissues were collected by the deer farm owner and placed into sterile Whirl-Pak bags and an aliquot of venous blood was collected. All the samples were maintained refrigerated until arrival at CHeRI on September 14, 2017. As the organ and tissue samples were collected > 24 h post-mortem and were decomposing, none were submitted for histopathology or microbiology analyses, though as previously noted, spleen tissue was used for virus isolation attempts as it had been collected < 48 h post-mortem.

**OV682.** On November 24, 2017, a previously healthy farmed 5-year old doe in Jefferson County, Florida (Figure 1), was found recumbent with its ears down and did not allow anyone to approach her. Later that day, the animal was unable to rise, unresponsive to stimuli, and died. A field necropsy was performed the following day by the deer farm owner, nearly 24 h after the animal had died, whereupon cardiac blood, heart, kidney, liver, lung, spleen, and small intestine tissues were removed and maintained refrigerated until delivery to CHeRI on November 30, 2017. Since necropsy was performed nearly 24 h post-mortem and the stage of decomposition somewhat advanced, no tissues were submitted for histopathology work. Lung tissue was submitted for diagnostic microbiology analyses, and spleen tissue was used for virus isolation attempts as it had been collected < 48 h post-mortem.

**OV862.** On September 18, 2018, a previously healthy farmed 1-year-old buck was found dead in Liberty County, FL (Figure 1). A necropsy was performed the same day by a CHeRI technician. Cardiac blood, and heart, kidney, liver, lung and spleen tissues were collected. As only the lung and kidney tissues appeared diseased, those were the only tissues submitted to the CHeRI for microbiology analyses. Spleen tissue was used for virus isolation attempts.

**OV867.** On September 20, 2018, a previously healthy farmed 3-month-old doe fawn in Duval County, Florida (Figure 1), was found struggling in the early morning, then died mid-morning. No signs of diseases had been observed the prior day. A necropsy was performed a few hours post-mortem by the farm owner, who only collected a kidney for microbiology analyses, and spleen tissue for virus isolation attempts.

**OV926.** On December 15, 2018, a previously healthy farmed 1-year-old white-tailed deer doe was found dead in Liberty County, Florida (Figure 1). No medications had been administered to the animal within the 3 months prior to death. A necropsy was performed on December 17, 2018, when the animal was at an advanced state of decomposition, and this precluded collection of tissues for diagnostic microbiology and histopathology. Spleen tissue was collected for virus isolation attempts.

### 2.3. Extraction of vgRNA from Virions in Blood and Tissue Homogenates for Initial RT-PCR Screens

Virus genomic RNA was extracted from virions in blood and tissue homogenates using a QIAamp Viral RNA mini kit (Qiagen, Valencia, California, USA) following the manufacturer’s protocol. SUPERase-In RNase inhibitor (Thermo Fisher Scientific, Waltham, Massachusetts, USA, Cat. Number AM2694) was added to the purified RNA samples, which were immediately stored at –80 °C to preserve them for molecular tests at a later time.

### 2.4. RT-PCR Screens for EHDV and BTV vgRNAs

RT-PCR screens for EHDV and BTV vgRNAs were performed following the protocol described by Ahasan, et al. [12]. Briefly, the RT-PCR multiplex assay for five viruses described by Wernike et al. [20] was modified for detection of BTV and EHDV vgRNAs only. Each individual RT-PCR reaction was performed in a total reaction volume of 25 μL using a VetMAX-Plus Multiplex One Step RT-PCR kit (Applied Biosystems, Foster City, California, USA). Individual reactions contained 12.5 μL 2X RT-PCR buffer, 2.5 μL 10X multiplex RT-PCR enzyme mix, 1 μL Xeno VIC Assay (which contains proprietary forward and reverse primers and a 5′ 2′-chloro-7′-phenyl-1,4-dichloro-6-carboxyfluorescein (VIC) flurophore and 3′ MGB (minor grove binder) NFQ (nonfluorescent quencher)–labeled probe, specific for an internal control RNA supplied with the kit), 0.1 μL of the Xeno RNA (internal control) (Applied Biosystems), 10 pmol of BTV forward and reverse primers, 2 pmol of BTV probe (fluorophore: 6-Carboxyfluorescein (6-FAM); quencher: Black Hole Quencher-1 (BHQ1), 15 pmol of EHDV forward and reverse primers, 2.5 pmol of EHDV probe (flurophore Texas Red; quencher BHQ2), and RNAse-free water to yield 20 μL master mix. After addition of 5 μL of template RNA, RT-PCR was performed in a 7500 fast Real-Time PCR System with the following thermal profile: reverse transcription step at 48 °C for 10 min, initial denaturation step at 95 °C for 10 min followed by 40 cycles of 3-step cycling consisting of denaturation at 95 °C for 15 s, annealing at 57 °C for 45 s, and final extension at 68 °C for 45 s.

### 2.5. RT-PCR Tests for Eastern Equine Encephalitis Virus (EEEV) and West Nile Virus (WNV) vgRNAs

RNA purified from spleen homogenate was also tested for EEEV and WNV vgRNAs, as the viruses are endemic in Florida and are known cervid pathogens. Both assays were carried out using the VetMAX Plus One Step RT-PCR kit (Applied Biosystems). Individual 25-μL reactions contained 12.5 μL 2X RT-PCR Buffer, 1 μL 25X RT-PCR enzyme, RNAase-free water, 1 μL Xeno VIC assay primers and probe, and 0.1 μL Xeno RNA (internal control), to which was added: (1) for WNV, 25 pmol of each primer and 5 pmol of probe: fluophore 6-FAM, quencher BHQ1, based on the procedure of Lanciotti et al. [21], or (2), for EEEV, 18 pmol of each primer and 5 pmol of probe: flurophore 6-FAM, quencher BHQ1, based on the procedure of Lambert et al. [22], yielding a 21-µL master-mix. Thereafter, 4 μL of template RNA was added to 21 μL of the master mix containing either WNV or EEEV specific primers and probe, and RT-PCRs performed in a 7500 fast Real-Time PCR System as follows: (1) for WNV, reverse transcription at 48 °C for 10 min, initial denaturation step at 95 °C for 10 min followed by 40 cycles of 2-step cycling consisting of 95 °C for 15 s and annealing/extension at 60 °C for 45 s, and (2) for EEEV, reverse transcription step at 48 °C for 10 min, initial denaturation step at 95 °C for 10 min followed by 40 cycles of 2-step cycling consisting of denaturation at 95 °C for 15 s and annealing/extension at 60 °C for 45 s.

### 2.6. RT-PCR Typing of EHDV

Presumptive determination of EHDV type was accomplished using RNA purified from spleen tissue following the method of Maan et al. [23]. Tests were performed with Seg-2 primers for discrimination between EHDV types 1, 2, or 6 only, as these are the typical EHDV types encountered in Florida and the rest of the United States. Each individual RT-PCR reaction was performed in a total reaction volume of 25 μL using a VetMAX-Plus Multiplex One Step RT-PCR kit (Applied Biosystems). Individual reactions contained 12.5 μL 2X RT-PCR buffer, 1.0 μL 25X multiplex RT-PCR enzyme mix, 1 μL Xeno VIC Assay primers and probe, and 0.1 μL Xeno RNA (internal control) (Applied Biosystems). For EHDV-1, each RT-PCR reaction contained 10 pmol of EHDV-1w/275F primer, EHDV-1w/341R primer, and 2.5 pmol of EHDV-1w/329P.probe, Similarly, for EHDV-2, each RT-PCR reaction contained 10 pmol each of EHDV-2w/1934F and EHDV-2w/2069R primers, and 2.5 pmol of EHDV-2w/1997P probe, and for EHDV-6, each RT-PCR reaction contained 10 pmol each of EHDV-6e/622F and EHDV-6e/724R primers and 2.5 pmol of EHDV-6e/669P probe. Probes for EHDV-1, -2, and -6 were 5′ labeled with 6-FAM flurophore and the quencher was BHQ1. To each EHDV-1, -2, and -6 reaction mixture, RNAse-free water was subsequently added to yield 20-μL master mixes. After addition of 5 μL of template RNA, RT-PCR was performed in a 7500 fast Real-Time PCR System with the following thermal profile: reverse transcription step at 50 °C for 10 min, initial denaturation step at 95 °C for 10 min followed by 50 cycles of 2-step cycling consisting of denaturation at 95 °C for 30 s and 60 °C degrees for 60 s.

### 2.7. Propagation of Cell Cultures for Virus Isolation

Cell lines used for virus isolation were obtained from the American Type Culture Collection (ATCC, Manassas, Virginia, USA). As BTV and EHDV were assumed to be the main agents of hemorrhagic disease in Florida’s farmed deer, virus isolation was attempted in two cell lines that are usually susceptible to and permissive for the viruses: C6/36 (*Aedes albopictus* (mosquito), ATCC CRL1660) and Vero E6 (*Cercopithecus aethiops* (African green monkey) kidney, ATCC CRL 1586). Both cell lines were grown as monolayers in a humidified atmosphere containing 5% CO_2_, the C6/36 cells at 28 °C and Vero E6 cells at 37 °C as described by Ahasan et al. [12].

### 2.8. Virus Isolation in Cultured Cells

Virus isolation attempts were made only from the spleens of animals OV610, OV617, OV862, and OV867 as they tested positive for EHDV-2 in primary RT-PCR screens (Table 1) and we thus assumed that virus was the causative agent of these animals’ deaths, and as mentioned above, EHDV can be isolated from spleen of white-tailed deer that have succumbed to the virus [8,11]. Similarly, EHDV was also considered the etiologic agent of OV926′s death based on gross examination of organs upon necropsy performed in the field. As OV926 was decomposing by the time of field necropsy, only the spleen was used for virus isolation attempts. For this animal, RT-PCR screens for EHDV lacked reproducibility; the first test generated a weak EHDV-specific positive PCR amplicon, but three follow-up tests generated negative results. In contrast, virus isolation attempts were made from blood, heart, kidney, liver, lung, small intestine, and spleen tissues obtained from animal OV682, as younger deer (3 months to 1 year old) are significantly more susceptible to BTV and EHDV, and this animal was 5 years old and there was a strong possibility that the etiologic agent was a virus other than BTV or EHDV. Moreover, BTV and EHDV vgRNAs were not detected by initial RT-PCR screens of RNA purified from its spleen, raising further suspicions that a different virus was the causative agent.

For all animals, virus isolation was attempted from tissue homogenates prepared as follows: portions of thawed tissues (heart, kidney, lung, liver, spleen, or small intestine) were aseptically weighed then homogenized in sterile phosphate-buffered saline (PBS) using a sterile manual tissue grinder (Fisher Scientific, Waltham, Massachusetts, USA) to form 10% (*w*/*v*) homogenates. After clearing of debris by low-speed centrifugation (5 min at 1500× *g*), the supernatants were filtered through a sterile 0.45 µm pore-size polyvinylidene fluoride filter (Fisher Scientific, Cat. Number 09-720-4) to remove bacteria and other particulates, and the resulting filtrates stored at −80 °C until further use. An aliquot of thawed whole blood from animal OV682 was also diluted 1:10, but was not filtered, for virus isolation attempts.

The 50-µL aliquots of filtered tissue homogenates and diluted blood from animal OV682 were inoculated onto subconfluent C6/36 and Vero E6 cells. Non-inoculated (mock-inoculated) cells were maintained in parallel as controls. The cells were re-fed every three days but monitored daily for mortality and virus-induced cytopathic effects (CPEs) for 30 days. In our work, if no CPEs are observed within the first 30 days, a second passage is routinely performed and the cells observed for another 30 days before being considered negative for virus isolation. Otherwise, after CPEs are observed in 50% of the inoculated C6/36 or Vero E6 cells, the infected cells are scraped and harvested along with the spent cell growth medium and stored at −80 °C for future analyses.

### 2.9. Sequencing of Virus Genomes

Virus identity and determination of the complete coding sequence (CDS) of all 10 genomes of the EHDVs and novel orbiviruses of this work were achieved through a next-generation sequencing (NGS) process as described previously [8,9,10,11,12]. Briefly, after approximately 50% of cultured cells inoculated with tissue homogenate displayed virus-induced CPEs, vgRNAs were extracted from virions in spent medium using a QIAamp viral RNA minikit (Qiagen). A cDNA library was thereafter constructed using a NEBNext Ultra RNA library prep kit (New England Biolabs, Ipswich, Massachusetts, USA) and sequenced on an Illumina MiSeq sequencer (Illumina, Inc., San Diego, California, USA). Details regarding the EHDV sequences obtained in this study will be included in a separate report to be presented elsewhere that presents a comprehensive analysis of EHDV genomes from dead Florida farmed deer that we have analyzed over the past three years.

### 2.10. Genome Assembly and Annotation of Virus Sequences

For each virus, paired-end reads obtained from the Illumina MiSeq were merged and quality trimmed in CLC Genomic Workbench v10.1.1 (Qiagen). As the viruses were isolated in C6/36 cells, sequence analyses were simplified by removal of host (*Aedes albopictus*) sequences deposited under GenBank accession number (GB number) MNAF00000000.2 by using Kraken v1.0 (Johns Hopkins University School of Medicine, Baltimore, Maryland, USA) [24]. Following removal of the host sequences, de novo assembly of pair-end reads was performed in SPAdes v3.5.0 (Center for Algorithmic Biotechnology, St. Petersburg State University, St. Pertersburg, Russia) with default parameters [25]. BLASTX (National Center for Biotechnology Information, U.S. National Library of Medicine, Bethesda, Maryland, USA) analysis of the assembled contigs were performed in CLC Genomic Workbench v10.1.1 against a custom virus genome database created from virus protein sequences retrieved from the UniProt Knowledgebase (https://www.uniprot.org/uniprot/). Contigs representing orbivirus genome segments were validated by mapping the quality- and host-filtered reads using Bowtie2 [26] and visually inspecting the alignments in Tablet 1.17.08.17 [27]. The average coverage of each viral genome was estimated using Qualimap v2.2.1. Putative open reading frames (ORFs) for the orbivirus genomes were predicted using GeneMarkS (http://exon.biology.gatech.edu/) [28] restricting the search to virus sequences. Additionally, the functions of the genes were predicted using BLASTP searches against the NCBI non-redundant protein database.

The GB numbers of the 10 CDSs of the six novel orbiviruses and four EHDVs of this report are listed in Table 2. In addition, for the six novel orbiviruses, information regarding the predicted gene functions of their respective segments are presented in Supplemental Table S1 (CHeRI OrbV 1.0, 2.1, and 2.2) and Supplemental Table S2 (CHeRI OrbV 3.1, 3.2, and 3.3).

### 2.11. Phylogenetic and Genetic Analyses

Maximum Likelihood (ML) phylogenetic analyses, based on the amino acid (aa) alignments of the RNA-dependent RNA polymerase (VP1) protein, the innermost sub-core capsid protein T2 (VP3), and outer capsid protein (VP2) of the CHeRI orbiviruses to those of 28 other orbiviruses whose sequences were available in GenBank (GB), were performed to assess their relationships. Amino acid sequences were aligned using (https://mafft.cbrc.jp/alignment/software/) MAFFT [29] and the ML analyses was performed in IQ-TREE-V 1.14.1 (http://www.iqtree.org/) with default settings and 1000 non-parametric bootstrap analyses to test the robustness of the hypothesis [30]. Phylogenetic trees were also constructed based on the nucleotide (nt) alignment of these genes (VP1, VP2 and VP3) following similar methods. Sequence identity matrices at both aa and nt levels were constructed using alignments of VP1, VP2, and VP3 (T2 protein) genes and proteins in Sequence Demarcation Tool v.1.2 using the MAFFT alignment option [31]. The GB numbers of the orbivirus protein and nt sequences that were analyzed are listed in Supplemental Table S3.

### 2.12. Transmission Electron Microscopy

As EHDV vgRNA was not detected by RT-PCR in a blood sample taken from animal OV682, transmission electron microscopy (TEM) of virus-infected C6/36 cells was performed to assist with virus identification before sequencing was attempted. Briefly, one nearly-confluent T75 Corning cell-culture treated flask (Thermo Fisher Scientific, Cat. number 7-202-000) of C6/36 cells was inoculated with 25 µL of first-passage cell lysate. The cells were fixed 4 days post-inoculation (dpi) in 15 mL of modified Karnovsky’s fixative (2P+2G, 2% formaldehyde prepared from paraformaldehyde and 2% glutaraldehyde in 0.1 M cacodylate buffer pH 7.4) at room temperature for 2 h, then the flask was stored at 4 °C for shipment to the University of Texas Medical Branch Electron Microscopy Laboratory (UTMB-EML). Prior to shipment on ice packs, the fixative was discarded, the cells washed once with 1 mL of ice-cold cacodylate buffer, then the wash buffer replaced with another 1 mL of ice-cold cacodylate buffer. The cells were subsequently scraped off the flask, then transferred along with the cacodylate buffer into a 15 mL conical tube, which was centrifuged at 3000× *g* for 10 min at 4 °C. After removing the cacodylate buffer, the pellet was resuspended in ice-cold PBS, and shipped to the UTMB-EML. At the UTMB-EML, the cell pellets were washed in cacodylate buffer and left in 2P+2G fixative overnight at 4 °C. The next day they were washed twice in cacodylate buffer, post-fixed in 1% OsO4 in 0.1 M cacodylate buffer pH 7.4, en bloc stained with 2% aqueous uranyl acetate, dehydrated in ascending concentrations of ethanol, processed through propylene oxide and embedded in Poly/Bed 812 epoxy plastic (Polysciences, Warrington, PA). Ultrathin sections (~75 nm) were cut on a Leica EM UC7 ultra-microtome (Leica Microsystems, Buffalo Grove, IL), stained with 0.4% lead citrate, and examined in a JEM-1400 electron microscope (JEOL USA, Peabody, MA) at 80 kV.

### 2.13. Diagnostic RT-PCR Tests Specific for CHeRI OrbV-1.0

CHeRI OrbV-1.0 was isolated in C6/36 cells from the spleen of animal OV682 but not from other tissue filtrates from the same animal. As virus isolation had initially been attempted only from spleen tissue, there was no indication whether animal OV682 was viremic for CHeRI OrbV-1.0. As a lack of CHeRI OrbV-1.0 isolation from specimens other than for spleen tissues (i.e., negative results) does not prove it was not present in the tissues that had been tested, and to gain insights on whether the virus was present in blood, two specific RT-PCR tests were devised based on the CHeRI OrbV-1.0 consensus sequences attained by NGS. Two separate RT-PCR tests were designed as a safeguard for future applications, considering gene reassortment occur among reoviruses, and mutations may occur at the RT-PCR primer-binding sites. CHeRI OrbV-1.0-specific RT-PCR tests were then used to screen aliquots of heart, kidney, liver, lung, and GI tract filtrates that had been archived at −80 °C, and frozen whole blood (Table 1). Briefly, a sequence within CHeRI OrbV-1.0 VP1 gene was the target of the first RT-PCR test. The primers for the VP1 gene RT-PCR assay (for both cDNA synthesis and PCR) consist of CHOrbV-1.F1 (forward; 5- GGAAGAAAATCTTGACAAGGTGAG-3) and CHOrbV-1.R1 (reverse; 5-CGTATTCAAGTATTTCTCGAACC-3), which generate a 335-base pair (bp) PCR amplicon. A sequence within the CHeRI OrbV-1 VP2 gene was the target of the second RT-PCR test. The primers for the VP2 gene RT-PCR assay (for both cDNA synthesis and PCR) consist of CHOrbV-1.F2 (forward: 5-GAGTGTGATAAAGATACAGGG-3) and CHOrbV-1.R2 (reverse; 5-CTTGTCCATCCTCAATATATCCC-3), which generate a 331-bp PCR amplicon. For both sets of CHeRI OrbV-1-specific primers, the same reverse transcription and PCR were performed as: After cDNA synthesis with Omniscript reverse transcriptase (Qiagen, Inc.) at 37 °C for 60 min using both F and R primers, PCR using both F and R primers was performed using OneTAQ DNA polymerase (New England Biolabs) using the following parameters: one cycle at 94 °C for 30 s, 35 cycles of 94 °C for 15 s, 47 °C for 30 s, and 68 °C for 40 s, and one cycle 68 °C for 4 min, terminated at 4 °C (∞).

### 2.14. Tropism of Virus Isolates in BHK-21 Cells

Because BHK-21 cells (*Mesocricetus auratus* (Syrian golden hamster) kidney cells) have historically been used in attempts at arbovirus isolation in cultured cells, we tested whether the viruses of this work could be propagated in them. When they were nearly confluent, BHK-21 cells (ATCC CCL-10) that had been propagated as monolayers at 37 °C in 5% CO_2_ in Advanced Minimum Essential Medium (aMEM, Invitrogen) supplemented 10% FBS, GlutaMAX, PSN, and 1X nonessential amino acids (Invitrogen) and a final concentration of 1 mM sodium pyruvate (Invitrogen) were inoculated with aliquots of first-passage virus isolated in C6/36 cells. The inoculated cells were re-incubated at 37 °C in 5% CO_2_ and observed for 30 days with re-feeds of the cells with reduced serum (3%) aMEM every three days before being considered negative for virus isolation. Virus isolation was based on formation of virus-induced CPEs and the detection of EHDV vgRNA in spent cell culture medium by RT-PCR. In the absence of CPEs, the spent media of cells inoculated with spleen homogenate from OV682 and OV926 were tested at 5 day intervals by RT-PCR for EHDV vgRNA, and at 15 dpi by NGS.

## 3. Results

### 3.1. Gross Pathology

The type of blood collected and key findings of the gross (macroscopic) examinations of tissues excised from the six dead deer of this report are given in Table 3. A common finding for all was the presence of external lung lesions, including hemorrhage in the lung tissues of four animals. The hearts of only two animals had a normal appearance: OV682 and OV926.

### 3.2. RT-PCR Screens of Blood and Spleen Tissue for BTV and EHDV vgRNAs

RT-PCR screens indicated EHDV-2 vgRNAs were present in the blood and spleens of animals OV610, -617, -862, and -867, but no BTV or other EHDV vgRNAs were detected in those specimens. In contrast, RT-PCR screens for BTV and EHDV vgRNAs in the blood and spleen were negative for specimens from OV682 and OV926 (Table 1).

### 3.3. Bacteriology and Mycology Cultures

As noted in Section 2.2, selected specimens from five of the six deer were submitted for microbiology (bacteriology and mycology) culture. The findings were unremarkable for all (i.e., for all the microbiology cultures, the findings were considered inconsequential and judged artefactual, related to post-mortem overgrowth by bacteria and fungi).

### 3.4. Histopathology

Histopathology analyses were performed for only one animal, OV610, as tissues collected from the other animals either displayed advanced autolysis upon necropsy and thus considered not suitable for anatomic diagnoses, or were not collected by the person performing the necropsy and gross pathology work. No pathologic processes resulting from infection by bacteria or fungi were revealed upon histologic evaluation of specimens taken from animal OV610.

### 3.5. Isolation of Virus Present in Spleen Filtrates

**Figure 2:**
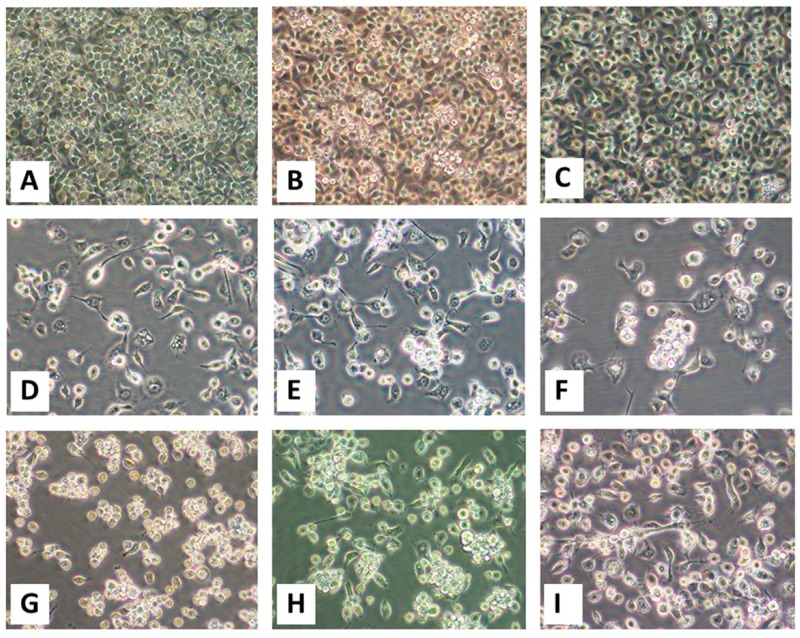
Virus-induced cytopathic effects (CPEs) in C6/36 cells. Panels (**A**–**C**): Mock-infected cells 3, 5, and 9 days post-inoculation (dpi) with phosphate-buffered saline (PBS). Cytopathic effects induced by the virus(es) stemming from spleen homogenates are shown for (**D**) OV610 at 9 dpi, (**E**) OV617 at 8 dpi, (**F**) OV682 at 9 dpi, (**G**) OV862 at 5 dpi, (**H**) OV867 at 4 dpi, and (**I**) OV926 at 9 dpi. All images were taken at an original magnification of 400×.

Virus was successfully isolated in C6/36 cells inoculated with aliquots of spleen homogenate from each of the six animals, and in Vero E6 from four of them (Table 1). For all, virus-induced CPEs were first observed in C6/36 cells, then several days later in Vero E6 cells. Initial CPEs in C6/36 cells consisted of the formation of cytoplasmic inclusions concomitant with granulation of the cytoplasm, followed by enlargement of the cells, some with fusiform morphology, then detachment and subsequent clumping of dead cells from the growing surface (Figure 2). Identical CPEs were observed in Vero E6 cells (data not shown). Observations specific for each specimen set (inoculated onto C6/36 and Vero cells) are indicated below according to animal:

**OV610.** Virus-induced CPEs were observed in C6/36 cells 6 dpi and in Vero E6 cells 15 dpi. Cytopathic effects formed by virus isolated from the spleen of OV610 in C6/36 cells 9 dpi are shown in Figure 2D.

**OV617.** Virus-induced CPEs were observed in C6/36 cells 7 dpi and in Vero E6 cells 23 dpi. Cytopathic effects formed by virus isolated from the spleen of OV617 in C6/36 cells 8 dpi are shown in Figure 2E.

**OV682.** Virus-induced CPEs were observed in C6/36 cells 5 dpi but were not observed in Vero E6 cells through 30 dpi. Cytopathic effects formed by virus isolated from the spleen of OV682 in C6/36 cells 9 dpi are shown in Figure 2F. In contrast, no CPEs were observed in C6/36 or Vero E6 cells inoculated with cardiac blood or with filtered homogenates prepared from heart, kidney, liver, lung, or GI tissues.

**OV862.** Virus-induced CPEs were observed in C6/36 cells 5 dpi and in Vero E6 cells 13 dpi. Cytopathic effects formed by virus isolated from the spleen of OV862 in C6/36 cells 5 dpi are shown in Figure 2G.

**OV867.** Virus-induced CPEs were observed in C6/36 cells 4 dpi and in Vero E6 cells 15 dpi. Cytopathic effects formed by virus isolated from the spleen of OV867 in C6/36 cells 4 dpi are shown in Figure 2H.

**OV926.** Virus-induced CPEs were observed in C6/36 cells 3 dpi but none were observed in Vero E6 cells through 30 dpi. Cytopathic effects formed by virus isolated from the spleen of OV926 in C6/36 cells 9 dpi are shown in Figure 2I.

### 3.6. Transmission Electron Microscopy of C6/36 Cells 4 dpi with Virus from Animal OV682

**Figure 3:**
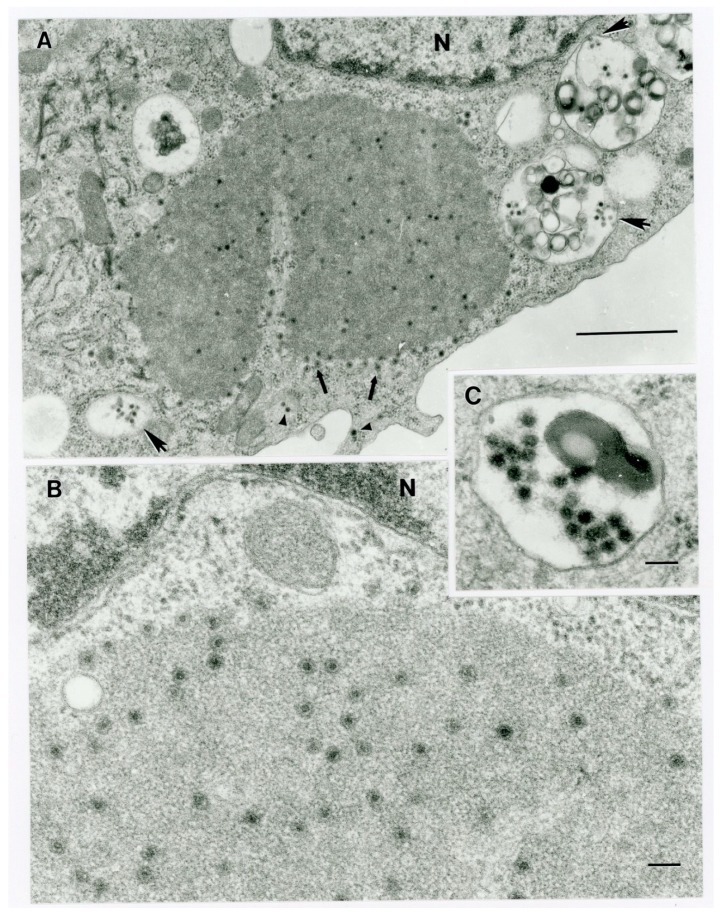
Ultrastructure of orbivirus OV682 in C6/36 cells. (**A**) Fragment of an infected cell with virus inclusion (“virus factory”) containing maturing virus particles (thick arrows) and virus-containing vacuoles (big arrowheads). Small arrowheads point out virus particles at the periphery of the cytosol. The letter N identifies a fragment of the host cell nucleus. Scale bar = 1 um. (**B**) Fragment of a cell demonstrating a portion of a virus factory with maturing particles. The letter N identifies a fragment of the host cell nucleus. Scale bar = 1 nm. (**C**) Fragment of an intracytoplasmic virus-containing vacuole. Scale bar = 1 nm.

Four dpi, virus particles in various states of maturation with diameters of 50–65 nm and with double-shelled capsids were present in cytoplasmic inclusions (“virus factories’, “viroplasms”) (Figure 3A,B) and intracellular vacuoles (Figure 3C). Close-up images of a cytoplasmic inclusion and a virus-containing vacuole from different host cells are shown in Figure 3B,C.

### 3.7. Complete Genome Sequencing, Assembly, and Genome Annotation of Novel Orbiviruses

Sequencing, assembly, and annotation details specific for the genomes of the novel orbiviruses isolated from each animal are given below. Details regarding the co-infecting EHDV-2s will be presented elsewhere as part of a large ongoing study of ours regarding EHDVs in circulation in Florida.

**Figure 4:**
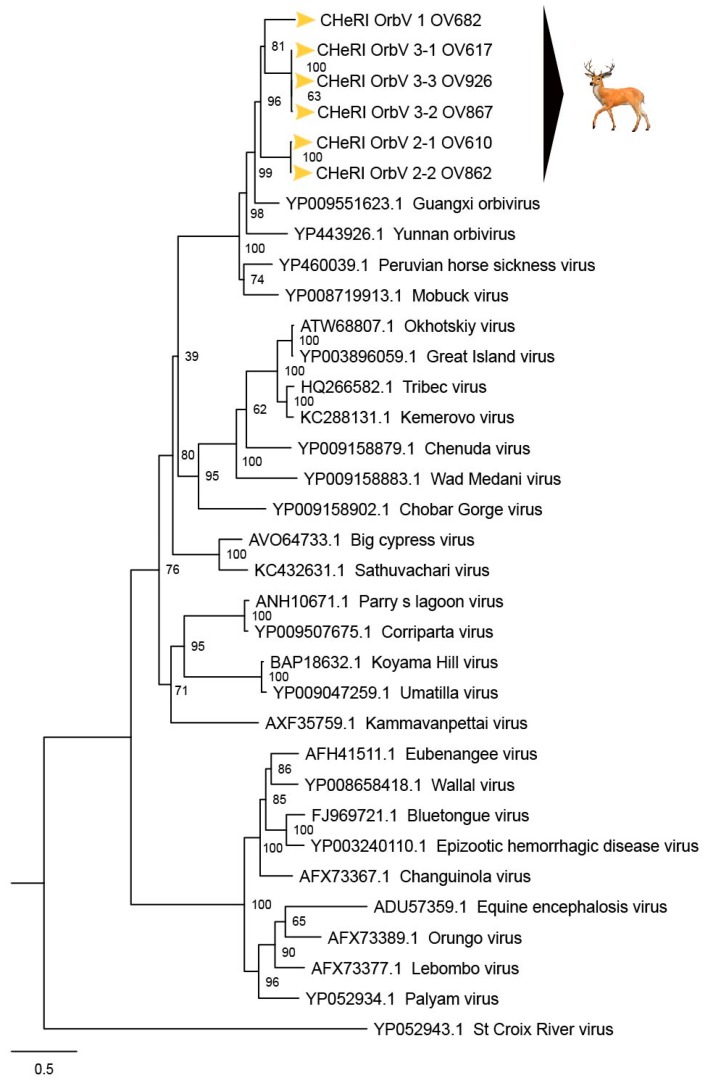
Maximum Likelihood (ML) phylogram of orbivirus T2 proteins. Shown is a ML phylogram depicting the relationship of the six CHeRI orbiviruses of this work to representatives of the genus *Orbivirus* based on the amino acid sequences of the T2 proteins coded by their innermost subcore capsid *VP3* genes. Bootstrap values are given at each node and the branch lengths represent the number of inferred substitutions as indicated by the scale.

**Figure 5:**
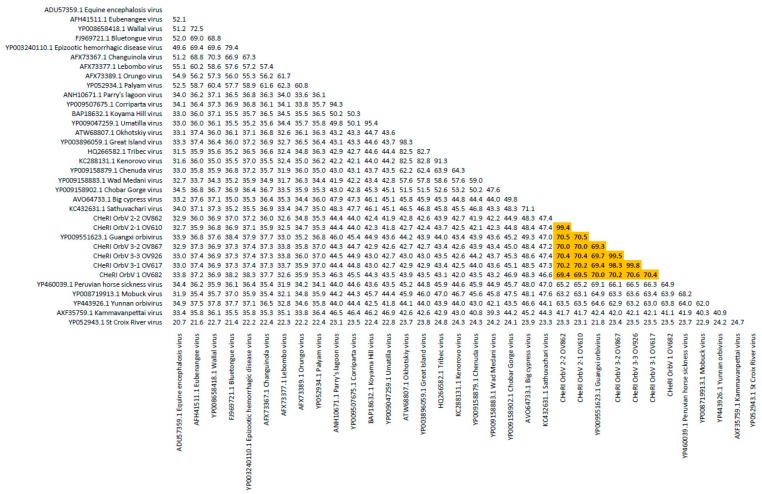
Sequence identity matrix of orbivirus T2 proteins. Shown is a sequence identity matrix depicting the amino acid percent identity of the six CHeRI orbiviruses of this work to 28 other orbiviruses based on the deduced amino acid sequences of the T2 proteins coded by their innermost subcore capsid *VP3* genes.

**OV610.** Illumina sequencing of the vgRNA purified from virions in the spent media of C6/36 cells resulted in a total of 3,518,466 pair-end reads, and a total of 3,518,262 high-quality reads. De novo assembly of the paired-end reads followed by BLASTX analyses recovered the coding sequences (CDS) for all 10 segments of an EHDV and another orbivirus. The deduced aa sequence of the major outer capsid VP2 protein of the EHDV (used for serotype determination) identified in the spleen of animal OV610 had highest aa identity (100%) with the VP2 aa sequence of an EHDV-2 (GB number AHC53345.1) that had previously been isolated from white-tailed deer in Pennsylvania in 2011. The novel orbivirus has been designated CHeRI orbivirus 2.1 (CHeRI OrbV 2.1) and its deduced T2 aa sequence had the closest identity (70.5%) with that of a Guangxi orbivirus (GXOV) isolated from cattle in China in 2015 (Figure 4 and Figure 5). The average depth of sequence coverage of the CHeRI OrbV-2.1 genome was 1920 reads/nucleotide (nt). The sizes of the 10 different CDS of CHeRI OrbV-2.1, their GB numbers, the inferred lengths of their structural and non-structural proteins, and the GB numbers of the 10 different CDS of the EHDV-2 virus from OV610 are listed in Supplemental Table S1.

**OV617.** Illumina sequencing of the vgRNA that had been purified from virions in the spent media of C3/36 cells resulted in a total of 1,557,806 pair-end reads, and a total of 1,556,401 high-quality reads. De novo assembly of the paired-end reads followed by BLASTX analyses recovered all 10 segments of an EHDV that is closely related (identity: 99.8%) to an EHDV-2 previously identified from white-tailed deer in Missouri, North America, in 2012 (GB number ALX38704.1). BLASTX analyses also recovered all 10 segments of a novel orbivirus whose deduced T2 aa sequence had highest identity (69.4%) to that of a GXOV isolated from cattle in China in 2015 (Figure 4 and Figure 5). This virus has been designated CHeRI OrbV 3-1. The average depth of sequence coverage of the CHeRI OrbV 3-1 genome was 1022 reads/nt. The sizes of the 10 different CDS segments of CHeRI OrbV 3-1, their GB numbers, and the inferred lengths of its structural and non-structural proteins, and the GB numbers of the 10 different CDS of the EHDV-2 virus from OV617, are listed in Supplemental Table S2.

**OV682.** Illumina sequencing of the vgRNA that had been purified from virions in the spent media of C3/36 cells resulted in a total of 3,492,822 pair-end reads, and a total of 3,492,238 high-quality reads. De novo assembly of the paired-end reads followed by BLASTX analyses recovered all 10 segments of a novel orbivirus and its deduced T2 aa sequence had the highest identity (70.0%) with that of a GXOV isolated from cattle in China in 2015 (Figure 4 and Figure 5). The virus has been designated CHeRI OrbV-1. The average depth of sequence coverage of the CHeRI OrbV-1 genome was 2485 reads/nt. The sizes of the 10 different CDS segments of CHeRI OrbV-1, its GB numbers, and the inferred lengths of its structural and non-structural proteins are listed in Supplemental Table S1.

**OV862.** Illumina sequencing of the vgRNA that had been purified from virions in the spent media of C3/36 cells resulted in a total of 1,557,806 pair-end reads, and a total of 1,556,401 high-quality reads. De novo assembly of the paired-end reads followed by BLASTX analyses recovered all 10 segments of an EHDV, and its deduced VP2 sequence suggests it is closely related (identity: 99.9%) to an EHDV-2 previously identified from white-tailed deer in Pennsylvania, North America in 2011 (GB number AHC53345.1). BLASTX analyses also recovered all 10 segments of a novel orbivirus whose deduced T2 aa sequence had highest aa identity to (70.5%) to that of a GXOV isolated from cattle in China in 2015 (Figure 4). This virus has been designated CHeRI OrbV-2-2. The average depth of sequence coverage of the CHeRI OrbV-2-2 genome was 361 reads/nt. The sizes of the 10 different CDS of CHeRI OrbV 2-2, their GB numbers, the inferred lengths of the structural and non-structural proteins, and the GB numbers of the 10 different CDSs of the EHDV-2 virus from OV862 are listed in Supplemental Table S1.

**OV867.** Illumina sequencing of the vgRNA that had been purified from virions in the spent media of C3/36 cells resulted in a total of 1,023,010 pair-end reads, and a total of 1,022,852 high quality reads were identified. De novo assembly of the paired-end reads followed by BLASTX analyses recovered all 10 segments of an EHDV. Analyses of its deduced VP2 aa sequence suggest this virus is closely related (identity: 99.1%) to an EHDV-2 strain previously identified in cattle in Indiana, USA, in 2007 (GB number AZS18673.1). BLASTX analyses also recovered all 10 segments of a novel orbivirus whose deduced T2 aa sequence had highest identity (72.1%) with that of a GXOV isolated from cattle in China in 2015 (Figure 4 and Figure 5). This orbivirus has been designated CHeRI OrbV-3-2. The average depth of sequence coverage of the CHeRI OrbV-3-2 genome was 472 reads/nt. The sizes of the 10 different CDS of CHeRI OrbV-3-2, their GB numbers, the inferred lengths of the structural and non-structural proteins, and the GB numbers of the 10 different CDS of the EHDV-2 virus from OV867 are listed in Supplemental Table S2.

**OV926.** Illumina sequencing of the vgRNA that had been purified from virions in the spent media of C3/36 cells resulted in a total of 2,343,370 pair-end reads, and a total of 2,343,136 high quality reads were identified after quality trimming in CLC Genomic Workbench 10.1.1 with default parameters. De novo assembly of the paired-end reads followed by BLASTX analyses recovered all 10 segments of a novel orbivirus, whose deduced T2 aa sequence had highest aa identity (69.7%) to that of a GXOV isolated from cattle in China in 2015 (Figure 4 and Figure 5). This virus has been designated CHeRI OrbV-3.3. The average coverage of the CHeRI OrbV-3.3 genome was 403 reads/nt. The sizes of the 10 different CDS of CHeRI OrbV-3.3, their GB numbers, and the inferred lengths of its structural and non-structural proteins, are listed in Supplemental Table S2.

### 3.8. Phylogenetic, Amino Acid, and Nucleotide Sequence Analyses of CHeRI Orbiviruses

**Figure 6:**
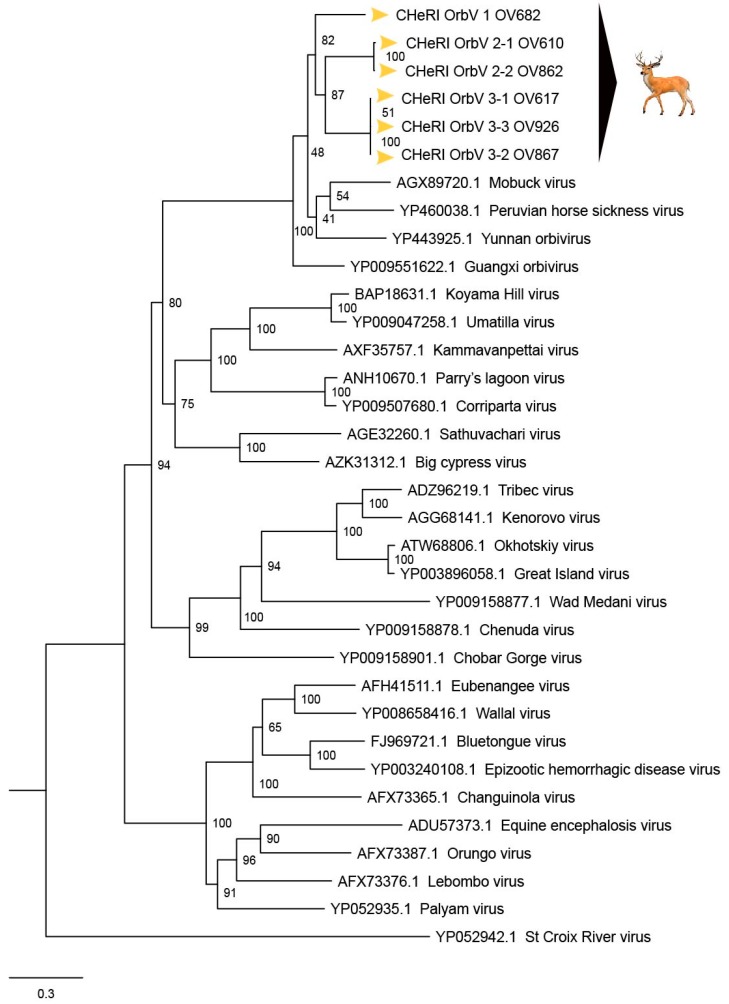
Maximum Likelihood (ML) phylogram of orbivirus RdRp proteins. Shown is a ML phylogram depicting the relationship of the six CHeRI orbiviruses of this work to representatives of the genus *Orbivirus* based on based on the deduced amino acid sequences of the RdRp proteins coded by their VP1 genes. Bootstrap values are given at each node and the branch lengths represent the number of inferred substitutions as indicated by the scale.

**Figure 7:**
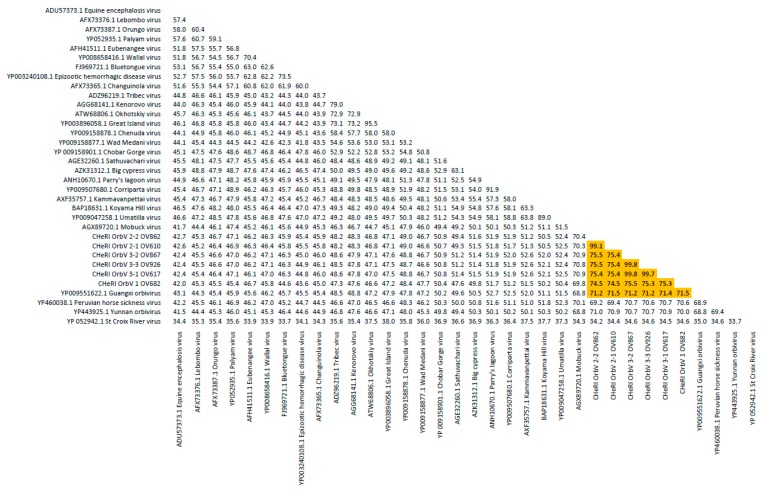
Sequence identity matrix of orbivirus RdRp proteins. Shown is a sequence identity matrix depicting the amino acid identity of the six CHeRI orbiviruses of this work to 28 other orbiviruses based on the deduced amino acid sequences of the RdRp proteins coded by their *VP1* genes.

**Figure 8:**
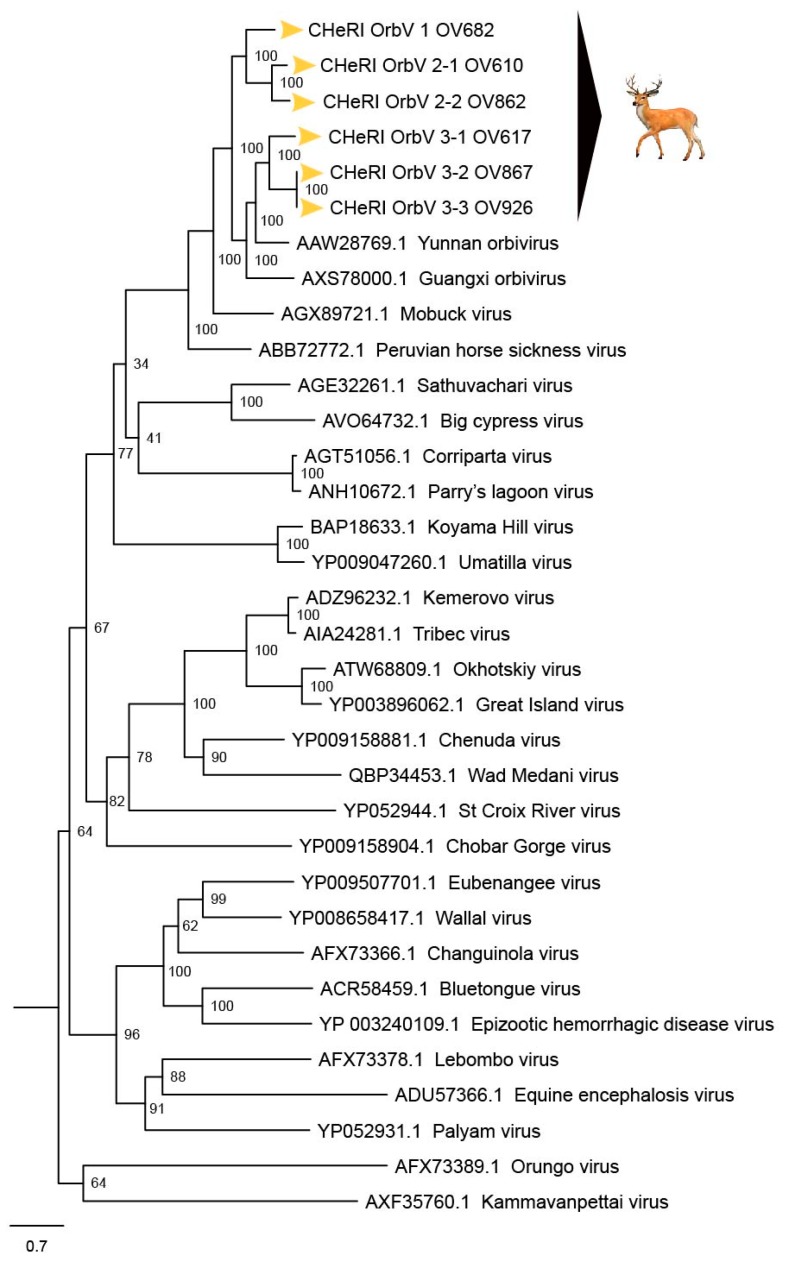
Maximum Likelihood (ML) phylogram of orbivirus major outer capsid proteins. Shown is a ML phylogram depicting the relationship of the six CHeRI orbiviruses of this work to representatives of the genus *Orbivirus* based on the deduced aa sequences of the major outer capsid proteins coded by their VP2 genes. Bootstrap values are given at each node and the branch lengths represent the number of inferred substitutions as indicated by the scale bar.

**Figure 9:**
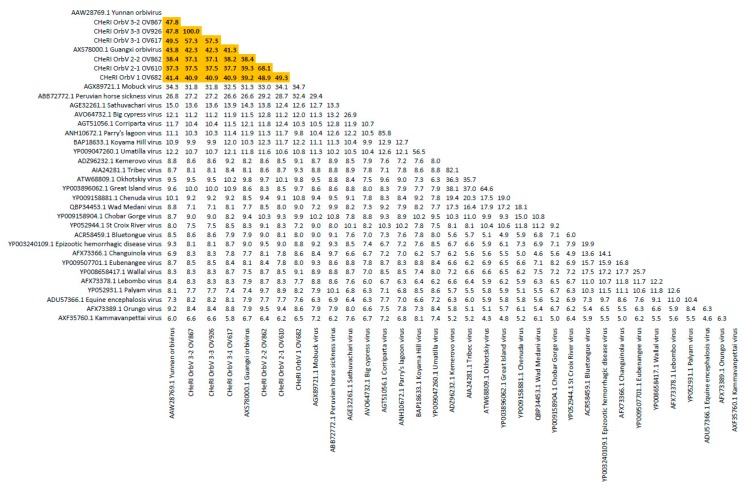
Sequence identity matrix of orbivirus major outer capsid proteins. Shown is a sequence identity matrix depicting the amino acid percentage identity of the six CHeRI orbiviruses of this work to 28 other orbiviruses based on the deduced amino acid sequences of the major outer capsid proteins coded by their *VP2* genes.

Phylogenetic and sequence identity matrix comparisons of the amino acid (aa) sequences of the deduced RdRp (VP1) proteins (Figure 6 and Figure 7), major outer capsid (VP2) proteins (Figure 8 and Figure 9), T2 (VP3) proteins (Figure 4 and Figure 5), and the nucleotide (nt) sequences of the VP1, VP2, and VP3 CDS (Figures S1–S6) of the six CHeRI orbiviruses compared to those of 28 other orbiviruses consistently group CHeRI OrbV-1, CHeRI OrbV-2.1, CHeRI OrbV-2.2, CHeRI OrbV-3.1, CHeRI OrbV-3.2 and CHeRI OrbV-3.3 with GXOV, Mobuck virus (MBV), PHSV, and Yunnan orbivirus (YUOV).

Phylogenetic analyses based on aa alignments of the RdRp proteins indicate the CHeRI isolates are highly related and branched as a sister clade to GXOV, MBV, PHSV, and YUOV, which are mosquito-borne orbiviruses (Figure 6). Genetic analyses reveal that the RdRp of the six CHeRI orbiviruses have highest aa identities (71.2–71.5%) to the RdRp of a GXOV isolated from cattle in China (Figure 7). CHeRI OrbV-1 displayed the maximum 70.6% aa identity, when compared to other CHeRI orbiviruses. CHeRI OrbV-2.1 displayed the highest (99.4%) aa identity to a CHeRI OrbV-2.2.

CHeRI OrbV 3.1 had the highest identities (98.3% and 98.8%) respectively, to CHeRI OrbV-3.2 and CHeRI OrbV-3.3 (Figure 7). The ML tree and genetic analysis at nt level revealed the CHeRI isolates as the closest relative to a GXOV isolated from cattle in China in 2015 (GB number NC_040478) (Figures S1 and S2). Taken together, the genetic analyses at both aa and nt levels supported CHeRI orbiviruses had > 30% (aa: 42.0–71.5%; nt: 41.8–69.1%) aa/nt identity to other known orbiviruses.

Maximum Likelihood analyses based on aa alignments of the T2 proteins supported all CHeRI isolates as strains of orbiviruses branching as the sister clade to GXOV isolated from cattle in China in 2015 (Figure 4). At the nt level, ML analyses of the *VP3* genes also revealed similar results: CHeRI isolates were the closest relative to a GXOV (GB number NC_040479), and a YUOV [30] isolated from mosquitoes (*Culex tritaeniorhynchus*) in China in 2005 (Figures S5 and S6).

Phylogenetic trees based on *VP2* gene aa/nt alignment suggest CHeRI orbiviruses as members of mosquito-borne orbivirus clade (Figure 8 and Figure S2). CHeRI OrbV-1 and CHeRI OrbV-2 branched as the sister clade to CHeRI OrbV-3 isolates while CHeRI OrbV-3 branched as sister clade to a YUOV. Genetic analysis revealed CHeRI OrbV-1 had the highest identity (aa: 49.3%; nt: 57.8%) to CHeRI OrbV-2.1, while CHeRI OrbV-2.1 displayed the closest identity (aa: 68.1%; nt: 69.3%) to CHeRI OrbV-2.2. CHeRI OrbV-3.1 had the highest identity (aa: 57.3%; nt: 60.5%) to CHeRI OrbV-3.2 and CHeRI OrbV-3.3, and CHeRI OrbV-3.2 was almost identical (aa/nt identity > 99.6%) to CHeRI OrbV-3.3 (Figure 8 and Figure S6).

### 3.9. Diagnostic RT-PCR Tests Specific for CHeRI OrbV-1.0

Among the tissues listed in Supplemental Table S4, the vRNA of CHeRI OrbV-1 could only be detected in nucleic acids extracted from cardiac blood and spleen tissues.

### 3.10. BHK-21 Tropism (Permissivity) Test

Virions in C6/36 cell lysates that tested positive for a novel orbivirus together with EHDV-2 formed CPEs in BHK-21 cells, as for Vero E6 cells. However, virions in the two C6/36 cell lysates that lacked a co-infecting EHDV-2 did not induce CPEs in BHK-21 cells, and neither of the novel orbiviruses (OrbV-1 and OrbV-3.3) nor EHDV-2 were detected by RT-PCR tests through 30 days post-inoculation (Table 1).

## 4. Discussion

Based on ICTV criteria for orbivirus classification [13], we reveal the discovery of three new orbivirus species along with three variants thereof in dead farmed white-tailed deer. The six novel reoviruses of this report fall within the genus *Orbivirus* since their deduced RdRp (VP1) aa sequences have > 30% identities with those of other orbiviruses such as GXOV, MBV, PHSV, and YUOV (see RdRp aa sequence identity matrix, Figure 7). Furthermore, according to ICTV criteria for orbivirus classification, T2 aa sequences are used to define orbivirus species; distinct species show < 91% sequence identities with the T2 sequences of other orbiviruses. Compared to other orbiviruses in Figure 5, the novel orbiviruses of this report comprise three new species: CHeRI OrbV-1, CHeRI OrbV-2, and CHeRI OrbV-3. Two variants of CHeRI OrbV-2 were identified: CHeRI OrbV-2.1, and CHeRI OrbV-2.2, as were three variants of CHeRI OrbV-3: CHeRI OrbV-3.1, CHeRI OrbV-3.2, and CHeRI OrbV-3.3 (see Maximum Likelihood Phylogram of orbivirus VP2 proteins, Figure 8). The six new orbiviruses share < 74% nt identity with other orbiviruses (Figure 9), and that fulfils the ICTV criteria for new species. Finally, TEM studies revealed that immature CHeRI OrbV-1 particles within C6/36 cells with diameters of 50–65 nm (Figure 3). This is consistent with observations for PHSV and YUOV, which form 55 to 60 nm immature virus particles within the cytoplasm of infected cells [32,33]. Taken together, the data indicate the viruses are undoubtedly orbiviruses.

Post-mortem lesions in white-tailed deer that die as a consequence of EHDV infections are characterized by hemorrhages and edema of varying severity and extent in multiple organs and tissues. Commonly affected sites include the heart and serosal surfaces of the pleural and peritoneal cavities; pulmonary edema and pericardial effusion is seen in some cases. Features suggestive of EHDV involvement were observed during gross examination of the deer of this report; in particular, lung involvement was common for all the dead deer (Table 1). However, it was specifically noted that the animals OV682 and OV926, from which were isolated CHeRI OrbV-1 and OrbV-3.3, respectively, but no co-infecting EHDV-2 or BTV (Table 1 and Table 3), had normal-appearing hearts. Future studies will have to address whether those viruses do not affect cardiac tissues.

No EEEV and WNV vgRNAs were detected in specimens from any of the six deer. Moreover, neither EEEV nor WNV were isolated in cell cultures inoculated with any of the specimens tested. While negative findings do not rule out the involvement of EEEV or WNV, the preponderance of the available evidence is consistent with an orbivirus infection leading to disease and death in the six animals. Phylogenetic evaluation of the viruses of this work (Figure 4, Figure 6 and Figure 8) consistently groups them with GXOV, MBV, PHSV, and YUOV. Mobuck virus was originally isolated from a white-tailed deer in Missouri, USA [34] and later from a white-tailed deer in Florida, North America [9]. The detection of viruses with high genetic relatedness to MBV in the dead white-tailed deer of this work appears consistent with the known biology of MBV. Guangxi virus, PHSV, and YUOV have been associated with disease in equids and cattle, and this raises the question whether the novel orbiviruses of this work are pathogens to those animals also. Another topic of interest resulting from this work is the question whether the new orbiviruses are a spill-over agents from cattle, equids, or some yet unspecified animal in Florida.

Due to the close genetic relatedness of the three new orbivirus species to the aforementioned mosquito-borne viruses, it is possible that the new viruses are also mosquito-borne viruses. Orbiviruses exemplified by YUOV usually can be isolated in mosquito (or other arthropod cells) but not in mammalian cells [32,33,34,35]. The six novel orbiviruses of this work were also not isolatable in Vero E6 or BHK-1 cells. These findings highlight the need to test other mammalian cell lines, especially bovine, equine and cervid cell lines and primary cells, for their utility as indicator cell lines for diagnostic work, and for basic research purposes. If these novel orbiviruses are found to contribute to disease, diagnostic labs need to incorporate them in their assessment of samples from moribund dear. Future development of diagnostic assays is needed to determine the prevalence of these orbiviruses in archived samples involving dear epizootics.

The prevention and control of arthropod-vectored orbiviruses is dependent upon knowledge of virus–vector–host interactions, pathogenesis, diagnostics, epidemiology, and control strategies. A better understanding of the basic virology of our new orbivirus is needed to: (1) help model current and future disease outbreaks, (2) develop vaccines, (3) develop reliable diagnostic tests, (4) develop effective vector control strategies, and (5) develop criteria and methods for domestic (enzootic) versus exotic (non-enzootic or incursive) serogrouping and topotyping. For our new viruses, transmission mechanisms in and between vertebrate hosts and arthropod vectors should be evaluated to determine their potential to emerge and threaten animal health, livestock trade, and even human health.

It is interesting that four of the six deer of this report had mixed virus infections. In each case, one of the novel orbiviruses was found together with EHDV-2. Each EHDV-2 strain was genetically distinct (the nucleotide sequences of their gene segments were not identical), indicating that the presence of EHDV-2 was not due to some sort of laboratory contaminant. Furthermore, we and others found MBV also in association with EHDV-2 [9,33]. An association of EHDV-2 with another orbivirus in infected tissue suggests some yet unknown interaction that may exacerbate pathology and warrants further investigation. According to our phylogenetic analyses, the novel orbiviruses group with mosquito-borne viruses whereas EHDV-2 is transmitted by *Culicoides* species. This raises the question whether our understanding of the vector biology of EHDV-2 is inaccurate (i.e., that the virus can also be vectored by mosquitoes), and vice-versa, whether the novel orbiviruses of this work are vectored not by mosquitoes but by *Culicoides* species. Additional research is needed to address these questions.

## 5. Conclusions

To our knowledge, these are the first observations/discoveries of infection by these six novel orbiviruses in white-tailed deer in the world. Continued surveillance efforts are needed to determine the potential threat these viruses may pose to the health of farmed and wild white-tailed deer populations and possibly to other animals.

## Figures and Tables

**Table 1:** List of key demographics and RT-PCR and virology findings.

Animal	Location (County, State)	Age	Sex	Tissues Tested	Virus-Induced Cytopathic Effects in Cultured Cells		RT-PCR Screens				EHDV Type	Detection and Confirmation of Virus in Cell Culture by Next-Generation Sequencing (NGS) and/or by RT-PCR	
					C6/36 Cells	VeroE6 Cells	BTV ^g^	EEEV ^h^	EHDV ^i^	WNV ^j^		C6/36 Cells	BHK-21 and VeroE6 Cells
OV610	Northumberland County, PA	2 years	M ^a^	blood	NT ^d^	NT	neg	neg	POS	neg	2	EHDV-2; CHeRI OrbV ^l^-2.1	EHDV-2
				spleen	POS ^e^	POS	neg	neg	POS	neg			
OV617	Jefferson county, FL	3 months	NS ^b^	blood	NT	NT	neg	neg	POS	neg	2	EHDV-2; CHeRI OrbV-3.1	EHDV-2
				spleen	POS	POS	neg	neg	POS	neg			
OV682	Jefferson county, FL	5 years	F ^c^	blood	NT	NT	neg	neg	neg	neg	NA ^k^	CHeRI OrbV-1.0	neg
				heart	neg ^f^	neg	NT	NT	NT	NT			
				kidney	neg	neg	NT	NT	NT	NT			
				liver	neg	neg	NT	NT	NT	NT			
				lung	neg	neg	NT	NT	NT	NT			
				GI tract	neg	neg	NT	NT	NT	NT			
				spleen	POS	neg	neg	neg	neg	neg			
OV862	Liberty County, FL	1 year	M	blood	NT	NT	neg	neg	POS	neg	2	EHDV-2; CHeRI OrbV-2.2	EHDV-2
				spleen	POS	POS	neg	neg	POS	neg			
OV867	Duval County, FL	3 months	F	blood	NT	NT	neg	neg	POS	neg	2	EHDV-2; CHeRI OrbV-3.2	EHDV-2
				spleen	POS	POS	neg	neg	POS	neg			
OV926	Liberty County, FL	1 year	F	blood	NT	NT	neg	neg	neg	neg	NA	CHeRI OrbV-3.3	neg
				spleen	POS	neg	neg	neg	neg	neg			

M ^a^, male; NS ^b^, not specified; F ^c^, female; NT ^d^, not tested; POS ^e^, positive for CPEs; neg ^f^, negative for CPEs; BTV ^g^*,* Bluetongue virus screen using RT-PCR assay of Wernike et al. [20]; EEEV ^h^, Eastern equine encephalitis virus screen using RT-PCR assay of Lambert et al. [22]; EHDV ^i^, Epizootic hemorrhagic disease virus screen using RT-PCR assay of Wernike et al. [20] and typing according to Maan et al. [23]; WNV ^j^, West Nile virus screen using RT-PCR assay of Lanciotti et al. [21]; NA ^k^, not applicable; CHeRI OrbV ^l^, CHeRI orbivirus.

**Table 2:** GenBank (GB) numbers of the 10 complete coding sequence (CDSs) of six novel orbiviruses and co-infecting EHDVs.

Animal	Strain Designation of Novel Orbivirus and Co-Infecting EHDV-2	Virus Genome Segment Number	Gene	GB Number Novel Orbivirus	GB Number EHDV-2
	CHeRI OrbV-2.1 and EHDV-2 strain OV610	1	*VP1*	MK903629	MK958987
		2	*VP3*	MK903631	MK958988
		3	*VP2*	MK903630	MK958989
		4	*VP4*	MK903632	MK958990
OV610		5	*NS1*	MK903636	MK958991
		6	*VP5*	MK903633	MK958992
		7	*NS2*	MK903637	MK958993
		8	*VP7*	MK903635	MK958994
		9	*VP6*	MK903634	MK958995
		10	*NS3*	MK903638	MK958996
	CHeRI OrbV-3.1 and EHDV-2 strain OV617	1	*VP1*	MK903649	MK958997
		2	*VP3*	MK903651	MK958998
		3	*VP2*	MK903650	MK958999
		4	*VP4*	MK903652	MK959000
OV617		5	*NS1*	MK903656	MK959001
		6	*VP5*	MK903653	MK959002
		7	*NS2*	MK903657	MK959003
		8	*VP7*	MK903655	MK959004
		9	*VP6*	MK903654	MK959005
		10	*NS3*	MK903658	MK959006
OV682	CHeRI OrbV-1 (no EHDV-2)	1	*VP1*	MK903619	(-) ^a^
		2	*VP3*	MK903621	(-)
		3	*VP2*	MK903620	(-)
		4	*VP4*	MK903622	(-)
		5	*NS1*	MK903626	(-)
		6	*VP5*	MK903623	(-)
		7	*NS2*	MK903627	(-)
		8	*VP7*	MK903625	(-)
		9	*VP6*	MK903624	(-)
		10	*NS3*	MK903628	(-)
OV862	CHeRI OrbV-2.2 and EHDV-2 strain OV682	1	*VP1*	MK903639	MK959007
		2	*VP3*	MK903641	MK959008
		3	*VP2*	MK903640	MK959009
		4	*VP4*	MK903642	MK959010
		5	*NS1*	MK903646	MK959011
		6	*VP5*	MK903643	MK959012
		7	*NS2*	MK903647	MK959013
		8	*VP7*	MK903645	MK959014
		9	*VP6*	MK903644	MK959015
		10	*NS3*	MK903648	MK959016
OV867	CHeRI OrbV-3.2 and EHDV-2 isolate OV687	1	*VP1*	MK903659	MK959017
		2	*VP3*	MK903661	MK959018
		3	*VP2*	MK903660	MK959019
		4	*VP4*	MK903662	MK959020
		5	*NS1*	MK903666	MK959021
		6	*VP5*	MK903663	MK959022
		7	*NS2*	MK903667	MK959023
		8	*VP7*	MK903665	MK959024
		9	*VP6*	MK903664	MK959025
		10	*NS3*	MK903668	MK959026
OV926	CHeRI OrbV-3.3 and EHDV-2 strain OV926	1	*VP1*	MK903669	(-)
		2	*VP3*	MK903671	(-)
		3	*VP2*	MK903670	(-)
		4	*VP4*	MK903672	(-)
		5	*NS1*	MK903676	(-)
		6	*VP5*	MK903673	(-)
		7	*NS2*	MK903677	(-)
		8	*VP7*	MK903675	(-)
		9	*VP6*	MK903674	(-)
		10	*NS3*	MK903678	(-)

(-) ^a^, no EHDV sequence detected by RT-PCR or NGS.

**Table 3:** Type of blood collected and gross examination findings.

Organ/Tissue	Animal Number					
	610	617	682	862	867	926
Blood	Cardiac blood	Venous blood	Cardiac blood	Cardiac blood	Cardiac blood	Cardiac blood
Heart	Adhesions, pale necrotic lesions, large amount of fluid in pericardial sac	Internally pale	Normal appearance	Dark red; evidence of pericarditis	Petechial hemorrhages on pericardium and heart surface; colored transudate in the pericardium	Normal appearance
Kidney	Advanced stage of decomposition	Normal appearance	Heavily covered in fat, dark purple exterior with dark interior	Moderate stage of decomposition	Light petechial hemorrhages on interior cut surface; minimal fat capsule	Not assessed
Liver	Dense	Normal appearance	Slight purple mottling on exterior	Normal appearance	Light, diffuse, mottled hemorrhage	Not assessed
Lung	Hemorrhaged; ante-mortem necrosis	Spongy; hemorrhaged; white foam present	Hemorrhaged	Very congested; spongy; foam present	Pale; diffuse hemorrhages on medial surface	Dark sections; foam present
Spleen	Viscid	Normal appearance	Very dark purple; gelatinous interior; hemorrhaged exterior; bloody	Normal appearance	Diffuse petechial hemorrhages	Advanced stage of decomposition
GI tract	Normal appearance	Not assessed	Normal appearance	Not assessed	Extensive external hemorrhages on portions of small and large intestines	Not assessed

## Supplementary Materials

The following are available online at https://www.mdpi.com/1999-4915/12/1/13/s1. Table S1. Lengths of CDSs and their encoded putative proteins, and GB numbers of CHeRI orbiviruses 1.0, 2.1, and 2.2. Table S2. Lengths of CDSs and their encoded putative proteins, and GB numbers of CHeRI orbiviruses 3.1, 3.2, and 3.3. Table S3. GenBank accession numbers of orbivirus protein acid and nucleotide sequences used for phylogenetic analyses. Table S4. Results of CHeRI OrbV-1-specific virus isolation and RT-PCR tests. Figure S1. Maximum Likelihood phylogram depicting the relationship of novel orbiviruses to representatives of the *Orbivirus* genus based on the nucleotide sequences of the gene RNA-dependent RNA polymerase, VP1. Figure S2. Sequence identity matrix showing the nucleotide percentage similarity of novel orbiviruses to 28 other orbiviruses based on the VP1 gene. Figure S3. Maximum Likelihood phylogram depicting the relationship of novel orbiviruses to representatives of the *Orbivirus* genus based on the nucleotide sequences of the gene for outer capsid protein VP2. Figure S4. Sequence identity matrix showing the nucleotide percentage similarity of novel orbiviruses to 28 other orbiviruses based on the VP2 gene. Figure S5. Maximum Likelihood phylogram depicting the relationship of novel orbiviruses to representatives of the *Orbivirus* genus based on the nucleotide sequences of the gene for innermost subcore capsid protein VP3 (T2). Figure S6. Sequence identity matrix showing the nucleotide percentage similarity of novel orbiviruses to 28 other orbiviruses based on the VP3 (T2) gene.
