# Supplementary material for: Three New Orbivirus Species Isolated from Farmed White-Tailed Deer (Odocoileus virginianus) in the United States
Source: Viruses. 2019 Dec 20;12(1):13. doi: 10.3390/v12010013 (PMC7019857; doi:10.3390/v12010013)
Supplement: Supplementary file 1 [file viruses-12-00013-s001.pdf]

**Table S1.** Lengths of CDSs and their encoded putative proteins, and GB nos. of CHeRI orbiviruses 1.0, 2.1, and 2.2.

| CHeRI OrbV | Parameter | Segment Number and Gene[c] | | | | | | | | | |
|---|---|---|---|---|---|---|---|---|---|---|---|
| | | 1 (VP1) | 2 (VP3) | 3 (VP2) | 4 (VP4) | 5 (NS1) | 6 (VP5) | 7 (NS2) | 8 (VP7) | 9 (VP6) | 10 (NS3) |
| 1.0 (from animal OV682) | CDS length (bp) including stop codon | 3,936 | 2,793 | 2,628 | 1,947 | 1,698 | 1,608 | 1,341 | 1,059 | 1,050 | 753 |
| | Predicted protein length (aa) | 1,311 | 930 | 875 | 648 | 565 | 535 | 446 | 352 | 349 | 250 |
| | GenBank accession number | MK903619 | MK903621 | MK903620 | MK903622 | MK903626 | MK903623 | MK903627 | MK903625 | MK903624 | MK903628 |
| 2.1[a] (from animal OV610) | CDS length (bp) including stop codon | 3,936 | 2,781 | 2,634 | 1,938 | 1,692 | 1,605 | 1,353 | 1,104 | 1,059 | 756 |
| | Predicted protein length (aa) | 1,311 | 926 | 877 | 645 | 563 | 534 | 450 | 367 | 352 | 251 |
| | GenBank accession number | MK903629 | MK903631 | MK903630 | MK903632 | MK903636 | MK903633 | MK903637 | MK903635 | MK903634 | MK903638 |
| 2.2[b] (from animal OV682) | CDS length (bp) including stop codon | 3,936 | 2,781 | 2,634 | 1,938 | 1,692 | 1,605 | 1,353 | 1,104 | 1,059 | 756 |
| | Predicted protein length (aa) | 1,311 | 926 | 877 | 645 | 563 | 534 | 450 | 367 | 352 | 251 |
| | GenBank accession number | MK903639 | MK903641 | MK903640 | MK903642 | MK903646 | MK903643 | MK903647 | MK903645 | MK903644 | MK903648 |

[a]GenBank accession numbers of the corresponding CDS of co-infecting EHDV-2 virus: MK958987 - MK958996. [b]GenBank accession numbers of the corresponding CDS of co-infecting EHDV-2 virus: MK959007 - MK959016. [c]Seg.1, VP1 gene, encodes RdRp; Seg.2, VP3 gene, encodes T2 protein; Seg.3, VP2 gene, encodes major outer capsid protein; Seg.4, VP4 gene, encodes Cap; Seg. 5, NS1 gene, encodes Tup; Seg.6, VP5, encodes outer capsid protein VP5; Seg.7, NS2 gene, encodes ssRNA-binding protein; Seg. 8, VP7 gene, encodes immunodominant major serogroup-specific antigen; Seg.9, VP6 gene, encodes NTPase (helicase); Seg.10, NS3 gene, encodes for protein involved in release of virus particles from infected insect cells.

**Table S2.** Lengths of CDSs and their encoded putative proteins, and GB nos. of CHeRI orbiviruses 3.1, 3.2, and 3.3.

| CHeRI OrbV | Parameter | Segment Number and Gene[c] | | | | | | | | | |
|---|---|---|---|---|---|---|---|---|---|---|---|
| | | 1 (VP1) | 2 (VP3) | 3 (VP2) | 4 (VP4) | 5 (NS1) | 6 (VP5) | 7 (NS2) | 8 (VP7) | 9 (VP6) | 10 (NS3) |
| 3.1[a] (from animal OV617) | CDS length (bp) including stop codon | 3,936 | 2,778 | 2,622 | 1,938 | 1,707 | 1,611 | 1,326 | 1,095 | 1,062 | 750 |
| | Predicted protein length (aa) | 1,311 | 925 | 873 | 645 | 568 | 536 | 441 | 364 | 353 | 249 |
| | GenBank accession number | MK903649 | MK903651 | MK903650 | MK903652 | MK903656 | MK903653 | MK903657 | MK903655 | MK903654 | MK903658 |
| 3.2[b] (from animal OV867) | CDS length (bp) including stop codon | 3,936 | 2,778 | 2,622 | 1,944 | 1,707 | 1,611 | 1,326 | 1,095 | 1,062 | 750 |
| | Predicted protein length (aa) | 1,311 | 925 | 873 | 647 | 568 | 536 | 441 | 364 | 353 | 249 |
| | GenBank accession number | MK903659 | MK903661 | MK903660 | MK903662 | MK903666 | MK903663 | MK903667 | MK903665 | MK903664 | MK903668 |
| 3.3 (from animal OV926 | CDS length (bp) including stop codon | 3,936 | 2,778 | 2,622 | 1,944 | 1,707 | 1,611 | 1,326 | 1,092 | 1,062 | 750 |
| | Predicted protein length (aa) | 1,311 | 925 | 873 | 647 | 568 | 536 | 441 | 363 | 353 | 249 |
| | GenBank accession number | MK903669 | MK903671 | MK903670 | MK903672 | MK903676 | MK903673 | MK903677 | MK903675 | MK903674 | MK903678 |

[a]GenBank accession numbers of the corresponding CDS of co-infecting EHDV-2 virus: MK958997 - MK959006. [b]GenBank accession numbers of the corresponding CDS of co-infecting EHDV-2 virus: MK959017 - MK959026. [c]Seg.1, VP1 gene, encodes RdRp; Seg.2, VP3 gene, encodes T2 protein; Seg.3, VP2 gene, encodes major outer capsid protein; Seg.4, VP4 gene, encodes Cap; Seg. 5, NS1 gene, encodes Tup; Seg.6, VP5, encodes outer capsid protein VP5; Seg.7, NS2 gene, encodes ssRNA-binding protein; Seg. 8, VP7 gene, encodes immunodominant major serogroup-specific antigen; Seg.9, VP6 gene, encodes NTPase (helicase); Seg.10, NS3 gene, encodes for protein involved in release of virus particles from infected insect cells.

**Table S3.** GenBank accession numbers of orbivirus protein acid and nucleotide sequences used for phylogenetic analyses.

| Orbivirus | VP1 (RdRp) | | VP2 (outer capsid protein) | | VP3 (T2) | |
|---|---|---|---|---|---|---|
| | Protein | Nucleotide | Protein | Nucleotide | Protein | Nucleotide |
| Big cypress orbivirus | AZK31312.1 | MK105769.1 | AVO64732.1 | MF094110.1 | AVO64733.1 | MF094111.1 |
| BTV | FJ969721.1 | FJ969719.1 | ACR58459.1 | FJ969720.1 | FJ969721.1 | FJ969721.1 |
| Changuinola virus | AFX73365.1 | JQ610655.1 | AFX73366.1 | JQ610656.1 | AFX73367.1 | JQ610657.1 |
| Chenuda virus | YP009158878.1 | NC_027534.1 | YP_009158881.1 | NC_027537.1 | YP009158879.1 | NC_027535.1 |
| CHeRI OrbV-1 OV682 | QCQ85336.1 | MK903619 | QCQ85337.1 | MK903620 | QCQ85338.1 | MK903621 |
| CHeRI OrbV-2.1 OV610 | QCQ85346.1 | MK903629 | QCQ85347.1 | MK903630 | QCQ85348.1 | MK903631 |
| CHeRI OrbV-2.2 OV862 | QCQ85356.1 | MK903639 | QCQ85357.1 | MK903640 | QCQ85358.1 | MK903641 |
| CHeRI OrbV-3.1 OV617 | QCQ85366.1 | MK903649 | QCQ85367.1 | MK903650 | QCQ85368.1 | MK903651 |
| CHeRI OrbV-3.2 OV926 | QCQ85376.1 | MK903659 | QCQ85377.1 | MK903660 | QCQ85378.1 | MK903661 |
| CHeRI OrbV-3.3 OV867 | QCQ85386.1 | MK903669 | QCQ85387.1 | MK903670 | QCQ85388.1 | MK903671 |
| Chobar Gorge virus | YP_009158901.1 | NC_027553.1 | YP_009158904.1 | NC_027556.1 | YP009158902.1 | NC_027554.1 |
| Corriparta virus | YP009507680.1 | NC_038568.1 | AGT51056.1 | KC853044.1 | YP009507675.1 | NC_038564.1 |
| EHDV | YP003240108.1 | NC_013396.1 | YP_003240109.1 | NC_013397.1 | YP003240110.1 | NC_013398.1 |
| Equine encephalosis virus | HQ630904.1 | ADU57359.1 | HQ630903.1 | ADU57366.1 | HQ630902.1 | ADU57373.1 |
| Eubenangee virus | JQ070378.1 | AFH41511.1 | NC_038588.1 | YP009507701.1 | JQ070376.1 | AFH41511.1 |
| Great Island virus | NC_014523.1 | YP003896059.1 | NC_014526.1 | YP_003896062.1 | NC_014522.1 | YP003896058.1 |
| Guangxi orbivirus | NC_040479.1 | YP009551623.1 | MG020126.1 | AXS78000.1 | NC_040478.1 | YP009551622.1 |
| Kammavanpettai virus | AXF35757.1 | MG770350.1 | AXF35760.1 | MG770353.1 | AXF35759.1 | MG770352.1 |
| Kemerovo virus | AGG68141.1 | KC288130.1 | ADZ96232.1 | HQ266594.1 | KC288131.1 | KC288131.1 |
| Koyama Hill virus | BAP18633.1 | AB894484.1 | BAP18633.1 | AB894486.1 | BAP18632.1 | AB894485.1 |
| Lebombo virus | AFX73376.1 | JQ610665.1 | AFX73378.1 | JQ610667.1 | AFX73377.1 | JQ610666.1 |
| Mobuck virus | AGX89720.1 | KF296322.1 | AGX89721.1 | KF296323.1 | YP008719913.1 | NC_022627.1 |
| Okhotskiy virus | ATW68806.1 | KY023329.1 | ATW68809.1 | KY023332.1 | ATW68807.1 | KY023330.1 |
| Orungo virus | AFX73387.1 | JQ610675.1 | AFX73389.1 | JQ610677.1 | AFX73389.1 | JQ610677.1 |
| Palyam virus | YP052935.1 | NC_005990.1 | YP052931.1 | NC_005986.1 | YP052934.1 | NC_005989.1 |
| Parry's lagoon virus | ANH10670.1 | KU724110.1 | ANH10672.1 | KU724112.1 | ANH10671.1 | KU724111.1 |
| PHSV | YP460038.1 | NC_007748.1 | ABB72772.1 | DQ248059.1 | YP460039.1 | NC_007749.1 |
| Sathuvachari virus | AGE32260.1 | KC432629.1 | AGE32261.1 | KC432630.1 | KC432631.1 | KC432631.1 |
| St Croix River virus | YP_052942.1 | NC_005997.1 | YP_052944.1 | NC_005999.1 | YP052943.1 | NC_005998.1 |
| Tribeč virus | ADZ96219.1 | HQ266581.1 | AIA24281.1 | KJ574045.1 | HQ266582.1 | HQ266582.1 |
| Umatilla virus | YP009047258.1 | NC_024503.1 | YP009047260.1 | NC_024505.1 | YP009047259.1 | NC_024504.1 |
| Wad Medani virus | YP009158877.1 | NC_027533.1 | QBP34453.1 | MH571967.1 | YP009158883.1 | NC_027539.1 |
| Wallal virus | YP008658416.1 | NC_022553.1 | YP_008658417.1 | NC_022554.1 | YP_008658418.1 | NC_022555.1 |
| Yunnan orbivirus | YP443925.1 | NC_007656.1 | AAW28769.1 | AY701511.1 | YP443926.1 | NC_007657.1 |

**Table S4.** Results of CHeRI OrbV-1-specific virus isolation and RT-PCR tests.

| Virus | Tissue | Virus isolation in: Vero E6 cells | Virus isolation in: C6/36 cells | RT-PCR |
|---|---|---|---|---|
| CHeRI OrbV-1 (from animal OV682) | cardiac blood | NT [a] | NT | POS |
| | heart | neg [b] | neg | neg |
| | kidney | neg | neg | neg |
| | liver | neg | neg | neg |
| | lung | neg | neg | neg |
| | spleen | neg | POS [c] | POS |
| | miscellaneous GI tract | neg | neg | neg |

NT [a], Not tested; neg [b], negative (no virus isolated); POS [c], Positive (virus isolated).

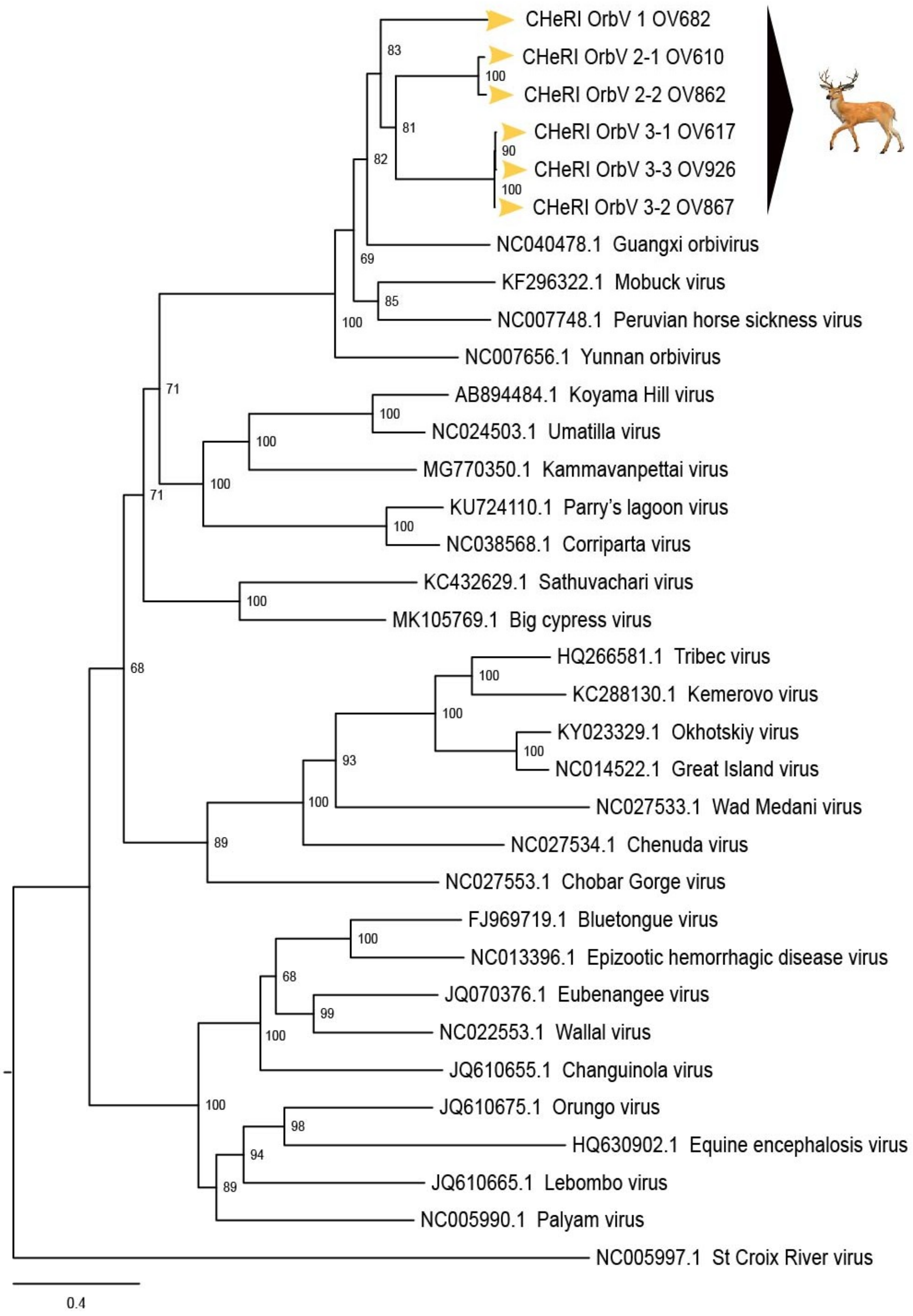


**Figure S1.** Maximum Likelihood phylogram depicting the relationship of novel orbiviruses to representatives of the *orbivirus* genus based on the nucleotide sequences of the gene RNA dependent RNA polymerase, VP1. Bootstrap values are given at each node and the branch lengths represent the number of inferred substitutions as indicated by the scale.

| | AB894484.1 Koyama Hill virus | NC 024503.1 Umatilla virus | MG770350.1 Kammavanpettai virus | KU724110.1 Parry's lagoon virus | NC 038568.1 Corriparta virus | KC432629.1 Sathuvachari virus | MK105769.1 Big cypress virus | CHeRI OrbV 1 OV682 | CHeRI OrbV 2-1 OV610 | CHeRI OrbV 2-2 OV862 | CHeRI OrbV 3-1 OV617 | CHeRI OrbV 3-2 OV867 | CHeRI OrbV 3-3 OV926 | NC040478.1 Guangxi orbivirus | KF296322.1 Mobuck virus | NC007748.1 Peruvian horse sickness virus | NC007656.1 Yunnan orbivirus | FJ969719.1 Bluetongue virus | NC013396.1 Epizootic hemorrhagic disease virus | JQ070376.1 Eubenangee virus | NC 022553.1 Wallal virus | JQ610655.1 Changuinola virus | JQ610665.1 Lebombo virus | JQ610675.1 Orungo virus | NC005990.1 Palyam virus | HQ630902.1 Equine encephalosis virus | HQ266581.1 Tribec virus | KC288130.1 Kemerovo virus | KY023329.1 Okhotskiy virus | NC014522.1 Great Island virus | NC027534.1 Chenuda virus | NC027533.1 Wad Medani virus | NC027553.1 Chobar Gorge virus | NC005997.1 St Croix River virus |
|---|---|---|---|---|---|---|---|---|---|---|---|---|---|---|---|---|---|---|---|---|---|---|---|---|---|---|---|---|---|---|---|---|---|---|
| AB894484.1 Koyama Hill virus | | | | | | | | | | | | | | | | | | | | | | | | | | | | | | | | | | |
| NC 024503.1 Umatilla virus | 76.1 | | | | | | | | | | | | | | | | | | | | | | | | | | | | | | | | | |
| MG770350.1 Kammavanpettai virus | 61.3 | 62.3 | | | | | | | | | | | | | | | | | | | | | | | | | | | | | | | | |
| KU724110.1 Parry's lagoon virus | 58.2 | 57.9 | 57.4 | | | | | | | | | | | | | | | | | | | | | | | | | | | | | | | |
| NC 038568.1 Corriparta virus | 56.5 | 57.1 | 57.5 | 78.1 | | | | | | | | | | | | | | | | | | | | | | | | | | | | | | |
| KC432629.1 Sathuvachari virus | 55.2 | 56 | 55.4 | 54.1 | 53.8 | | | | | | | | | | | | | | | | | | | | | | | | | | | | | |
| MK105769.1 Big cypress virus | 55.6 | 57.3 | 56.4 | 55.3 | 55.8 | 60.4 | | | | | | | | | | | | | | | | | | | | | | | | | | | | |
| CHeRI OrbV 1 OV682 | 54.6 | 56.2 | 55.8 | 55.5 | 55.2 | 53.5 | 54.9 | | | | | | | | | | | | | | | | | | | | | | | | | | | |
| CHeRI OrbV 2-1 OV610 | 55.3 | 56.4 | 56.8 | 56.5 | 55.8 | 54.9 | 56.9 | **71.6** | | | | | | | | | | | | | | | | | | | | | | | | | | |
| CHeRI OrbV 2-2 OV862 | 55.2 | 56.3 | 56.7 | 56.5 | 55.7 | 54.7 | 56.8 | **71.7** | **96.1** | | | | | | | | | | | | | | | | | | | | | | | | | |
| CHeRI OrbV 3-1 OV617 | 55.7 | 56.1 | 56.4 | 55.1 | 55.8 | 54 | 56.2 | **69.9** | **71.3** | **71.1** | | | | | | | | | | | | | | | | | | | | | | | | |
| CHeRI OrbV 3-2 OV867 | 55.8 | 56 | 56.4 | 55.1 | 55.7 | 54 | 56.1 | **70.1** | **71.2** | **71.2** | **99.3** | | | | | | | | | | | | | | | | | | | | | | | |
| CHeRI OrbV 3-3 OV926 | 55.6 | 55.9 | 56.4 | 54.9 | 55.7 | 53.9 | 56.2 | **69.9** | **71.2** | **71.1** | **99.3** | **99.3** | | | | | | | | | | | | | | | | | | | | | | |
| NC040478.1 Guangxi orbivirus | 55.6 | 56.1 | 55.4 | 55.8 | 55.4 | 54.2 | 55 | **68.4** | **69.3** | **69.1** | **68.1** | **68.2** | **68.2** | | | | | | | | | | | | | | | | | | | | | |
| KF296322.1 Mobuck virus | 54.4 | 55.6 | 55.2 | 54.3 | 53.7 | 54 | 54 | 67.2 | 67.7 | 67.8 | 66.7 | 66.7 | 66.6 | 66.5 | | | | | | | | | | | | | | | | | | | | |
| NC007748.1 Peruvian horse sickness virus | 55.7 | 56.3 | 55.4 | 55.2 | 54.3 | 54.5 | 55.7 | 67.5 | 68.7 | 68.1 | 67.7 | 67.7 | 67.7 | 66.7 | 67.3 | | | | | | | | | | | | | | | | | | | |
| NC007656.1 Yunnan orbivirus | 54.6 | 55.5 | 55.1 | 54.2 | 53.5 | 54.1 | 55 | 66.2 | 67 | 66.8 | 66.6 | 66.4 | 66.5 | 65.6 | 65.7 | 66.4 | | | | | | | | | | | | | | | | | | |
| FJ969719.1 Bluetongue virus | 51.5 | 52 | 51.7 | 51.4 | 50.7 | 50.9 | 51.9 | 51.9 | 52.5 | 52.2 | 51.4 | 51.6 | 51.5 | 52 | 51.2 | 52.2 | 51.7 | | | | | | | | | | | | | | | | | |
| NC013396.1 Epizootic hemorrhagic disease virus | 51.4 | 52.8 | 51.8 | 51.1 | 51.2 | 50.6 | 52.1 | 51.8 | 52.6 | 52.7 | 51.6 | 51.7 | 51.8 | 52.2 | 52.1 | 52.7 | 51.7 | 67.5 | | | | | | | | | | | | | | | | |
| JQ070376.1 Eubenangee virus | 51.1 | 51.5 | 52.3 | 51.6 | 51.5 | 51.6 | 52.6 | 51.8 | 52.5 | 52.6 | 51.8 | 51.8 | 52.1 | 51.8 | 51.8 | 52.3 | 51.6 | 62 | 62.2 | | | | | | | | | | | | | | | |
| NC 022553.1 Wallal virus | 52.7 | 52.8 | 54.1 | 52.2 | 52.4 | 51.6 | 53 | 53.3 | 54.3 | 54.4 | 53.9 | 53.8 | 53.9 | 53.6 | 53 | 53.7 | 52.5 | 61.6 | 63.1 | 66.3 | | | | | | | | | | | | | | |
| JQ610655.1 Changuinola virus | 51 | 52.5 | 52.3 | 51.2 | 51.1 | 51.2 | 53.7 | 52.7 | 53.4 | 53.3 | 53.3 | 53.3 | 53.3 | 52.2 | 51.8 | 52.5 | 52.1 | 61.3 | 60.1 | 61.3 | 63.6 | | | | | | | | | | | | | |
| JQ610665.1 Lebombo virus | 51.3 | 51.5 | 52.1 | 51 | 51.5 | 51.1 | 52.1 | 51.2 | 50.6 | 51.2 | 50.3 | 50.1 | 50.2 | 50 | 49.3 | 51.4 | 50.2 | 57.6 | 58.4 | 57.7 | 57.5 | 56.7 | | | | | | | | | | | | |
| JQ610675.1 Orungo virus | 51.9 | 52.4 | 51.7 | 51.4 | 52.1 | 52.1 | 52.3 | 51.1 | 52.4 | 52.3 | 51.8 | 51.9 | 51.9 | 50.7 | 51.1 | 51.6 | 51.6 | 57.5 | 56.4 | 57.3 | 57.4 | 57 | 59.7 | | | | | | | | | | | |
| NC005990.1 Palyam virus | 52.8 | 53.3 | 52.7 | 52.7 | 52.4 | 52.2 | 53.1 | 52.3 | 53.3 | 53.4 | 52.7 | 52.7 | 52.6 | 52.9 | 52.3 | 53.1 | 52 | 56.8 | 57.4 | 57.6 | 59.5 | 58.8 | 59.4 | 59.7 | | | | | | | | | | |
| HQ630902.1 Equine encephalosis virus | 52.1 | 51.3 | 50.8 | 50.5 | 50.2 | 50.2 | 51.7 | 49.2 | 49.7 | 49.5 | 49.8 | 49.7 | 49.7 | 49.7 | 49.3 | 50.3 | 48.6 | 55.9 | 54.8 | 53.9 | 55.2 | 53.8 | 55.7 | 56.6 | 56.1 | | | | | | | | | |
| HQ266581.1 Tribec virus | 51.4 | 50.1 | 50.1 | 51.3 | 50.1 | 49.8 | 51.6 | 49.1 | 49.3 | 49.5 | 49 | 49.2 | 49.2 | 49.5 | 47.9 | 49.5 | 49.8 | 48.7 | 49.5 | 48.7 | 47.4 | 47.2 | 49.4 | 50.4 | 48.1 | 48.4 | | | | | | | | |
| KC288130.1 Kemerovo virus | 50.4 | 50 | 48.9 | 50.8 | 50.8 | 49.7 | 51.2 | 49.8 | 49.1 | 49.3 | 49.2 | 49.3 | 49.3 | 49.6 | 47.7 | 50.2 | 49.2 | 48.2 | 48.8 | 47.8 | 48 | 48.3 | 50 | 49.4 | 48.4 | 48.1 | 69.9 | | | | | | | |
| KY023329.1 Okhotskiy virus | 50.1 | 49.7 | 49 | 49.7 | 49.4 | 49.4 | 49.9 | 48 | 47.7 | 47.7 | 49.1 | 49.3 | 49.1 | 48 | 47.7 | 48.4 | 48.5 | 48.2 | 48.3 | 48.1 | 47.2 | 47 | 49.1 | 49.5 | 47.8 | 48.5 | 65.7 | 65.5 | | | | | | |
| NC014522.1 Great Island virus | 50.7 | 50.3 | 49.9 | 50.5 | 50.7 | 50.2 | 51.3 | 49.3 | 48.4 | 48.5 | 49.6 | 49.7 | 49.6 | 48.2 | 47.6 | 48.8 | 48.8 | 48.5 | 48.5 | 48.9 | 47.2 | 48.2 | 49.2 | 49.9 | 47.7 | 47.8 | 66.5 | 65.2 | 82.6 | | | | | |
| NC027534.1 Chenuda virus | 51.1 | 50.9 | 50.1 | 51.5 | 51.7 | 50.4 | 50.5 | 50.5 | 49.2 | 49.7 | 50 | 50.1 | 50.1 | 49.1 | 49 | 49.8 | 49.4 | 48.7 | 48.8 | 48.9 | 47.7 | 47.3 | 49.1 | 50 | 48.8 | 47.9 | 56.1 | 56.2 | 55.7 | 55.9 | | | | |
| NC027533.1 Wad Medani virus | 50.4 | 49.6 | 49.5 | 50 | 49.7 | 49.7 | 50.2 | 49.6 | 48.3 | 48.4 | 48.9 | 49.2 | 48.9 | 48.2 | 49.4 | 48.9 | 48.5 | 48.3 | 47.4 | 48.2 | 47.5 | 47.3 | 49.1 | 49 | 47.4 | 48.4 | 54.5 | 54.1 | 53.3 | 53.7 | 53.5 | | | |
| NC027553.1 Chobar Gorge virus | 51.9 | 51.1 | 51 | 51.8 | 52.2 | 51.9 | 52.6 | 51.6 | 51.3 | 51.4 | 52.6 | 52.6 | 52.6 | 51.4 | 50.7 | 51.1 | 51.6 | 50.2 | 49.8 | 51.1 | 50.4 | 48.7 | 50.6 | 49.6 | 50.1 | 48.9 | 52.9 | 52.5 | 53.4 | 53.4 | 52.7 | 51.7 | | |
| NC005997.1 St Croix River virus | 43.3 | 43.1 | 42.9 | 42.9 | 42.3 | 43.2 | 43.9 | 42.6 | 42.3 | 42.4 | 41.7 | 41.8 | 41.8 | 42.4 | 42.3 | 42.2 | 42 | 42.1 | 42.2 | 42.2 | 41.6 | 42 | 43.3 | 43.8 | 42.4 | 42.6 | 43.1 | 42.3 | 43.6 | 44.4 | 42.5 | 43.2 | 43.2 | |

**Figure S2.** Sequence identity matrix showing the nucleotide percentage similarity of novel orbiviruses to 28 other orbiviruses based on the VP1 gene.

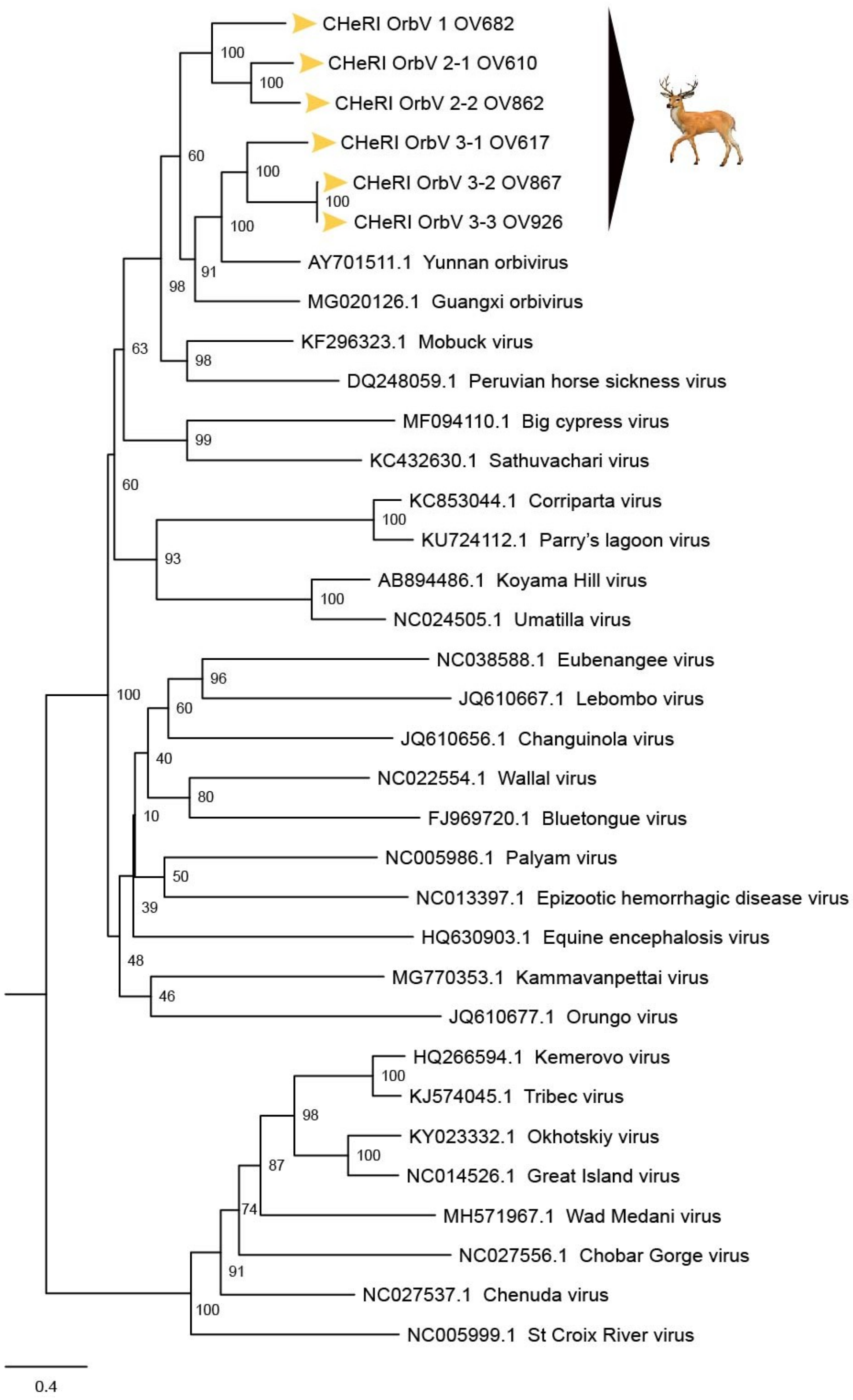


**Figure S3.** Maximum Likelihood phylogram depicting the relationship of novel orbiviruses to representatives of the *orbivirus* genus based on the nucleotide sequences of the gene for outer capsid protein VP2. Bootstrap values are given at each node and the branch lengths represent the number of inferred substitutions as indicated by the scale.

| | AB894486.1 Koyama Hill virus | NC 024505.1 Umatilla virus | AY701511.1 Yunnan orbivirus | CHeRI OrbV 3-1 OV617 | CHeRI OrbV 3-2 OV867 | CHeRI OrbV 3-3 OV926 | CHeRI OrbV 1 OV682 | CHeRI OrbV 2-1 OV610 | CHeRI OrbV 2-2 OV862 | MG020126.1 Guangxi orbivirus | DQ248059.1 Peruvian horse sickness virus | KF296323.1 Mobuck virus | MF094110.1 Big cypress virus | NC022554.1 Wallal virus | NC005986.1 Palyam virus | KC432630.1 Sathuvachari virus | JQ610656.1 Changuinola virus | NC038588.1 Eubenangee virus | NC013397.1 Epizootic hemorrhagic disease virus | HQ630903.1 Equine encephalosis virus | FJ969720.1 Bluetongue virus | KC853044.1 Corriparta virus | KU724112.1 Parry's lagoon virus | JQ610667.1 Lebombo virus | MG770353.1 Kammavanpettai virus | JQ610677.1 Orungo virus | HQ266594.1 Kemerovo virus | KJ574045.1 Tribec virus | KY023332.1 Okhotskiy virus | NC014526.1 Great Island virus | NC027537.1 Chenuda virus | MH571967.1 Wad Medani virus | NC005999.1 St Croix River virus | NC027556.1 Chobar Gorge virus |
|---|---|---|---|---|---|---|---|---|---|---|---|---|---|---|---|---|---|---|---|---|---|---|---|---|---|---|---|---|---|---|---|---|---|---|
| AB894486.1 Koyama Hill virus | | | | | | | | | | | | | | | | | | | | | | | | | | | | | | | | | | |
| NC 024505.1 Umatilla virus | 60.0 | | | | | | | | | | | | | | | | | | | | | | | | | | | | | | | | | |
| AY701511.1 Yunnan orbivirus | 28.5 | 29.5 | | | | | | | | | | | | | | | | | | | | | | | | | | | | | | | | |
| CHeRI OrbV 3-1 OV617 | 29.4 | 29.7 | 57.2 | | | | | | | | | | | | | | | | | | | | | | | | | | | | | | | |
| CHeRI OrbV 3-2 OV867 | 28.2 | 28.1 | 55.4 | 60.5 | | | | | | | | | | | | | | | | | | | | | | | | | | | | | | |
| CHeRI OrbV 3-3 OV926 | 28.2 | 28.2 | 55.5 | 60.5 | 99.6 | | | | | | | | | | | | | | | | | | | | | | | | | | | | | |
| CHeRI OrbV 1 OV682 | 30.2 | 29.0 | 50.4 | 51.1 | 49.9 | 50.0 | | | | | | | | | | | | | | | | | | | | | | | | | | | | |
| CHeRI OrbV 2-1 OV610 | 28.7 | 28.5 | 49.4 | 49.5 | 49.1 | 49.1 | 57.8 | | | | | | | | | | | | | | | | | | | | | | | | | | | |
| CHeRI OrbV 2-2 OV862 | 29.3 | 28.5 | 50.6 | 49.9 | 48.8 | 48.9 | 56.5 | 69.3 | | | | | | | | | | | | | | | | | | | | | | | | | | |
| MG020126.1 Guangxi orbivirus | 29.1 | 28.4 | 52.0 | 51.5 | 50.9 | 51.0 | 50.0 | 51.2 | 50.0 | | | | | | | | | | | | | | | | | | | | | | | | | |
| DQ248059.1 Peruvian horse sickness virus | 28.7 | 28.2 | 42.9 | 44.3 | 43.0 | 43.0 | 46.0 | 46.3 | 46.1 | 43.2 | | | | | | | | | | | | | | | | | | | | | | | | |
| KF296323.1 Mobuck virus | 28.1 | 28.5 | 45.4 | 47.1 | 45.5 | 45.8 | 46.3 | 46.3 | 46.4 | 44.8 | 44.2 | | | | | | | | | | | | | | | | | | | | | | | |
| MF094110.1 Big cypress virus | 23.4 | 23.0 | 28.6 | 28.7 | 27.4 | 27.4 | 28.7 | 29.2 | 28.6 | 27.1 | 28.4 | 30.2 | | | | | | | | | | | | | | | | | | | | | | |
| NC022554.1 Wallal virus | 23.5 | 23.3 | 30.6 | 31.0 | 30.4 | 30.4 | 31.6 | 30.4 | 30.3 | 29.7 | 29.0 | 28.7 | 24.6 | | | | | | | | | | | | | | | | | | | | | |
| NC005986.1 Palyam virus | 22.7 | 22.3 | 28.6 | 28.8 | 28.6 | 28.5 | 28.8 | 30.2 | 29.3 | 28.6 | 27.9 | 29.5 | 23.1 | 26.8 | | | | | | | | | | | | | | | | | | | | |
| KC432630.1 Sathuvachari virus | 23.7 | 24.6 | 31.0 | 31.3 | 30.2 | 30.2 | 30.0 | 31.7 | 31.0 | 31.6 | 29.7 | 30.6 | 25.7 | 25.5 | 24.2 | | | | | | | | | | | | | | | | | | | |
| JQ610656.1 Changuinola virus | 21.3 | 21.1 | 25.7 | 24.8 | 25.6 | 25.6 | 25.8 | 26.3 | 25.1 | 25.7 | 25.1 | 25.1 | 22.2 | 27.6 | 26.6 | 21.2 | | | | | | | | | | | | | | | | | | |
| NC038588.1 Eubenangee virus | 23.9 | 23.5 | 28.8 | 29.0 | 29.3 | 29.3 | 29.7 | 29.5 | 29.3 | 29.9 | 29.8 | 27.7 | 23.5 | 27.8 | 27.3 | 23.7 | 25.8 | | | | | | | | | | | | | | | | | |
| NC013397.1 Epizootic hemorrhagic disease virus | 22.3 | 23.0 | 26.8 | 27.6 | 27.8 | 27.7 | 27.8 | 29.5 | 28.9 | 27.5 | 29.5 | 26.7 | 24.6 | 26.4 | 27.1 | 25.0 | 26.3 | 26.0 | | | | | | | | | | | | | | | | |
| HQ630903.1 Equine encephalosis virus | 21.8 | 21.9 | 26.6 | 26.0 | 26.9 | 27.0 | 26.4 | 27.4 | 28.0 | 25.8 | 24.8 | 25.8 | 22.7 | 25.2 | 25.4 | 22.7 | 25.4 | 25.2 | 24.5 | | | | | | | | | | | | | | | |
| FJ969720.1 Bluetongue virus | 23.2 | 22.7 | 27.5 | 28.3 | 28.1 | 28.1 | 28.5 | 28.1 | 27.3 | 27.3 | 27.9 | 26.5 | 22.7 | 28.5 | 26.2 | 23.6 | 27.5 | 26.6 | 25.7 | 24.2 | | | | | | | | | | | | | | |
| KC853044.1 Corriparta virus | 24.4 | 25.1 | 25.6 | 26.4 | 25.9 | 26.0 | 25.9 | 25.9 | 25.9 | 24.4 | 25.4 | 26.0 | 22.0 | 21.7 | 22.0 | 23.4 | 20.7 | 22.2 | 21.2 | 19.3 | 21.8 | | | | | | | | | | | | | |
| KU724112.1 Parry's lagoon virus | 24.1 | 24.5 | 24.8 | 25.3 | 24.8 | 24.7 | 25.1 | 25.4 | 25.2 | 24.2 | 25.1 | 25.5 | 22.5 | 22.1 | 21.1 | 23.1 | 20.3 | 22.8 | 21.6 | 18.9 | 21.7 | 74.7 | | | | | | | | | | | | |
| JQ610667.1 Lebombo virus | 22.0 | 22.3 | 26.9 | 27.7 | 27.7 | 27.8 | 26.6 | 28.0 | 26.9 | 26.7 | 26.6 | 28.0 | 21.0 | 25.1 | 24.7 | 22.0 | 25.6 | 28.3 | 24.2 | 24.5 | 23.4 | 21.8 | 19.9 | | | | | | | | | | | |
| MG770353.1 Kammavanpettai virus | 21.6 | 21.5 | 22.4 | 22.0 | 23.2 | 23.2 | 23.1 | 22.9 | 23.5 | 22.2 | 21.0 | 22.2 | 20.3 | 19.7 | 20.0 | 20.8 | 17.1 | 20.1 | 19.0 | 17.6 | 20.9 | 21.5 | 21.3 | 18.5 | | | | | | | | | | |
| JQ610677.1 Orungo virus | 21.3 | 20.5 | 26.6 | 28.0 | 26.2 | 26.2 | 26.5 | 27.8 | 26.8 | 27.0 | 26.2 | 26.1 | 21.2 | 24.5 | 25.1 | 22.0 | 22.1 | 25.2 | 23.1 | 22.0 | 23.3 | 20.4 | 20.6 | 22.5 | 18.9 | | | | | | | | | |
| HQ266594.1 Kemerovo virus | 17.5 | 17.7 | 18.0 | 18.3 | 18.7 | 18.7 | 18.9 | 18.5 | 19.4 | 18.1 | 17.4 | 18.8 | 17.1 | 15.3 | 15.3 | 17.3 | 13.4 | 16.0 | 15.1 | 13.6 | 15.2 | 14.5 | 15.3 | 14.5 | 13.7 | 13.9 | | | | | | | | |
| KJ574045.1 Tribec virus | 17.2 | 17.5 | 17.6 | 17.8 | 18.2 | 18.1 | 18.4 | 18.6 | 18.3 | 17.2 | 17.8 | 19.0 | 16.7 | 15.2 | 14.4 | 17.4 | 13.2 | 14.9 | 14.3 | 13.6 | 14.3 | 14.1 | 15.0 | 14.6 | 13.7 | 13.2 | 75.3 | | | | | | | |
| KY023332.1 Okhotskiy virus | 17.3 | 17.3 | 18.0 | 17.8 | 17.9 | 18.0 | 18.6 | 18.5 | 18.6 | 17.8 | 17.9 | 18.8 | 17.3 | 14.5 | 15.4 | 17.1 | 12.5 | 15.4 | 14.8 | 14.6 | 14.2 | 15.4 | 16.2 | 15.1 | 13.6 | 13.2 | 47.1 | 46.8 | | | | | | |
| NC014526.1 Great Island virus | 17.8 | 17.9 | 18.4 | 18.6 | 18.4 | 18.4 | 18.7 | 18.7 | 18.2 | 17.9 | 18.2 | 18.3 | 17.7 | 14.8 | 15.1 | 17.3 | 13.8 | 16.0 | 14.6 | 14.7 | 14.1 | 15.5 | 15.8 | 14.8 | 14.0 | 13.8 | 48.7 | 47.8 | 64.5 | | | | | |
| NC027537.1 Chenuda virus | 18.0 | 17.5 | 19.5 | 20.1 | 19.3 | 19.3 | 19.3 | 19.5 | 19.5 | 18.2 | 19.5 | 19.8 | 16.4 | 15.6 | 14.8 | 16.9 | 14.9 | 15.9 | 14.7 | 14.0 | 16.2 | 14.8 | 15.7 | 15.5 | 14.4 | 14.5 | 38.9 | 39.3 | 37.8 | 37.5 | | | | |
| MH571967.1 Wad Medani virus | 18.7 | 19.4 | 19.6 | 19.2 | 18.6 | 18.6 | 18.8 | 18.6 | 17.9 | 19.3 | 18.3 | 19.3 | 16.8 | 15.3 | 14.7 | 16.8 | 13.8 | 15.9 | 14.5 | 14.6 | 15.9 | 16.4 | 16.1 | 14.4 | 14.6 | 14.1 | 37.1 | 37.1 | 37.5 | 36.8 | 34.2 | | | |
| NC005999.1 St Croix River virus | 17.7 | 16.7 | 21.4 | 20.7 | 20.4 | 20.5 | 21.5 | 20.4 | 20.4 | 20.0 | 20.0 | 20.4 | 18.4 | 16.2 | 17.1 | 19.6 | 14.7 | 17.8 | 16.7 | 15.4 | 16.2 | 17.5 | 17.3 | 16.1 | 14.4 | 16.4 | 28.1 | 27.1 | 27.8 | 27.4 | 28.1 | 29.6 | | |
| NC027556.1 Chobar Gorge virus | 18.1 | 16.1 | 17.3 | 17.9 | 18.1 | 18.1 | 18.7 | 18.4 | 18.2 | 18.0 | 17.2 | 17.7 | 15.6 | 15.5 | 15.0 | 17.0 | 13.5 | 15.4 | 14.4 | 14.7 | 14.5 | 14.4 | 15.2 | 14.1 | 13.5 | 13.3 | 30.5 | 29.6 | 30.1 | 30.3 | 30.6 | 28.8 | 25.5 | |

**Figure S4.** Sequence identity matrix showing the nucleotide percentage similarity of novel orbiviruses to 28 other orbiviruses based on the VP2 gene.

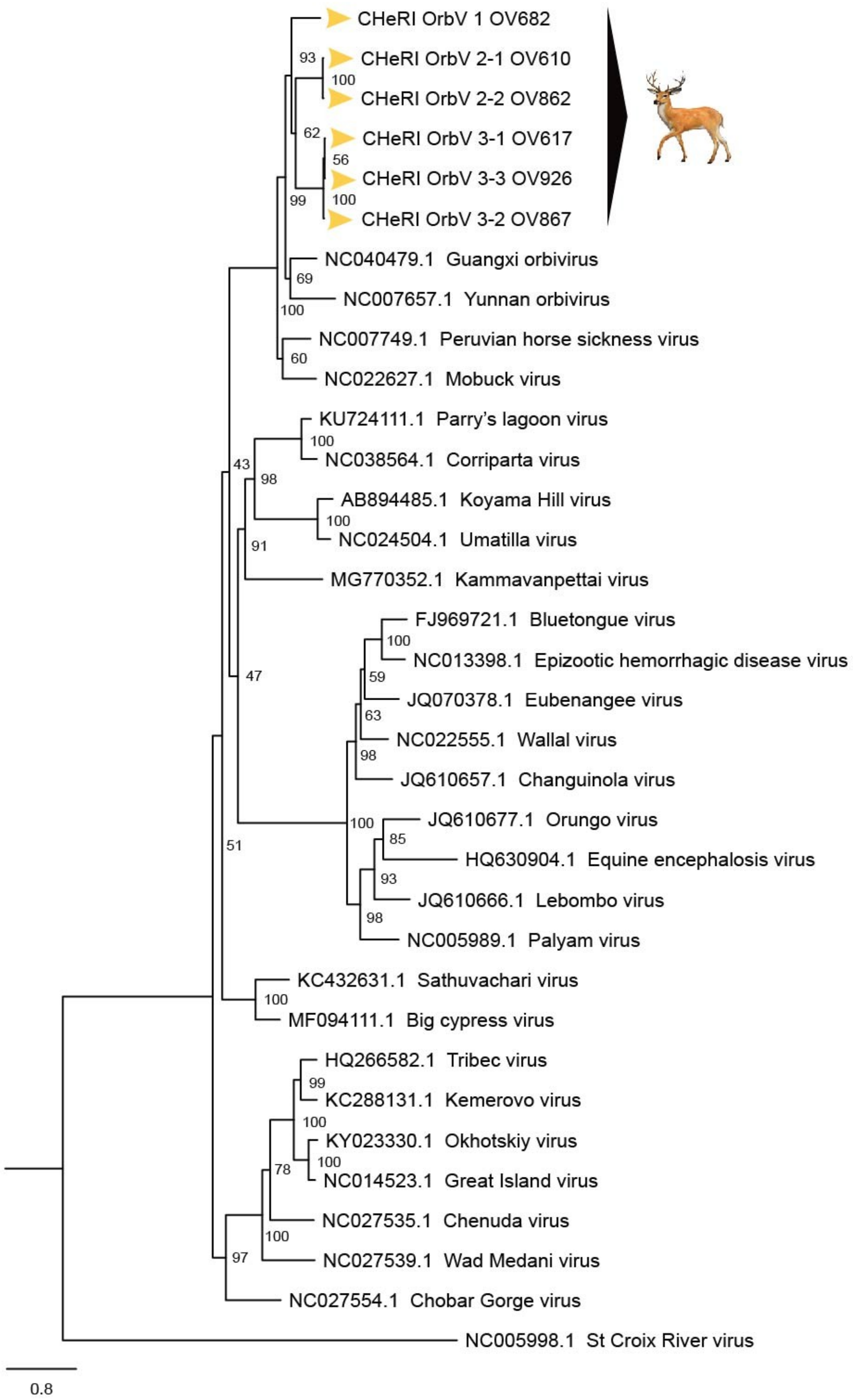


**Figure S5.** Maximum Likelihood phylogram depicting the relationship of novel orbiviruses to representatives of the *orbivirus* genus based on the nucleotide sequences of the gene for innermost subcore capsid protein VP3 (T2). Bootstrap values are given at each node and the branch lengths represent the number of inferred substitutions as indicated by the scale.

| | AB894485.1 Koyama Hill virus | NC024504.1 Umatilla virus | KU724111.1 Parry's lagoon virus | NC038564.1 Corriparta virus | CHeRI OrbV 1 OV682 | CHeRI OrbV 2-1 OV610 | CHeRI OrbV 2-2 OV862 | CHeRI OrbV 3-1 OV617 | CHeRI OrbV 3-3 OV926 | CHeRI OrbV 3-2 OV867 | NC040479.1 Guangxi orbivirus | NC007749.1 Peruvian horse sickness virus | NC022627.1 Mobuck virus | NC007657.1 Yunnan orbivirus | KC432631.1 Sathuvachari virus | MF094111.1 Big cypress virus | NC027554.1 Chobar Gorge virus | HQ266582.1 Tribec virus | KC288131.1 Kemerovo virus | KY023330.1 Okhotskiy virus | NC014523.1 Great Island virus | NC027535.1 Chenuda virus | NC027539.1 Wad Medani virus | MG770352.1 Kammavanpettai virus | FJ969721.1 Bluetongue virus | NC013398.1 Epizootic hemorrhagic disease virus | JQ070378.1 Eubenangee virus | NC022555.1 Wallal virus | JQ610657.1 Changuinola virus | NC005989.1 Palyam virus | JQ610666.1 Lebombo virus | JQ610677.1 Orungo virus | HQ630904.1 Equine encephalosis virus | NC005998.1 St Croix River virus |
|---|---|---|---|---|---|---|---|---|---|---|---|---|---|---|---|---|---|---|---|---|---|---|---|---|---|---|---|---|---|---|---|---|---|---|
| AB894485.1 Koyama Hill virus | | | | | | | | | | | | | | | | | | | | | | | | | | | | | | | | | | |
| NC024504.1 Umatilla virus | 79 | | | | | | | | | | | | | | | | | | | | | | | | | | | | | | | | | |
| KU724111.1 Parry's lagoon virus | 54.6 | 55.4 | | | | | | | | | | | | | | | | | | | | | | | | | | | | | | | | |
| NC038564.1 Corriparta virus | 55.3 | 54.9 | 79.9 | | | | | | | | | | | | | | | | | | | | | | | | | | | | | | | |
| CHeRI OrbV 1 OV682 | 53.3 | 52.3 | 54.4 | 53.5 | | | | | | | | | | | | | | | | | | | | | | | | | | | | | | |
| CHeRI OrbV 2-1 OV610 | 50.7 | 51.4 | 53.2 | 52.2 | **68.4** | | | | | | | | | | | | | | | | | | | | | | | | | | | | | |
| CHeRI OrbV 2-2 OV862 | 50.4 | 51.2 | 53.1 | 52.1 | **68** | **98.1** | | | | | | | | | | | | | | | | | | | | | | | | | | | | |
| CHeRI OrbV 3-1 OV617 | 50.6 | 51 | 52.8 | 51.9 | **67.3** | **68.5** | **68.6** | | | | | | | | | | | | | | | | | | | | | | | | | | | |
| CHeRI OrbV 3-3 OV926 | 50.8 | 51.1 | 53 | 52.1 | **67.6** | **68.7** | **68.8** | **98.8** | | | | | | | | | | | | | | | | | | | | | | | | | | |
| CHeRI OrbV 3-2 OV867 | 50.6 | 50.8 | 53 | 52 | **67.9** | **68.9** | **68.9** | **97.2** | **97.3** | | | | | | | | | | | | | | | | | | | | | | | | | |
| NC040479.1 Guangxi orbivirus | 52 | 51.8 | 53.1 | 52.5 | **68.2** | **67.3** | **67.5** | **66.1** | **66.1** | **65.7** | | | | | | | | | | | | | | | | | | | | | | | | |
| NC007749.1 Peruvian horse sickness virus | 51.8 | 52.5 | 53.1 | 52.4 | **65.2** | **64.9** | **64.7** | **66.9** | **66.9** | **67** | **65.8** | | | | | | | | | | | | | | | | | | | | | | | |
| NC022627.1 Mobuck virus | 52.4 | 51.8 | 52.7 | 52.3 | 64 | 63.7 | 64.2 | 64.2 | 64.2 | 64.5 | 64.4 | 66.9 | | | | | | | | | | | | | | | | | | | | | | |
| NC007657.1 Yunnan orbivirus | 50.8 | 50.7 | 52.8 | 53 | 64.1 | 63.1 | 63.1 | 62 | 62.2 | 62.5 | 64.1 | 62.6 | 62.3 | | | | | | | | | | | | | | | | | | | | | |
| KC432631.1 Sathuvachari virus | 51.6 | 52 | 53.6 | 52.9 | 52.4 | 53.3 | 53.4 | 52.5 | 52.6 | 52.6 | 53.6 | 54.9 | 53.2 | 50.2 | | | | | | | | | | | | | | | | | | | | |
| MF094111.1 Big cypress virus | 53 | 53.1 | 54.7 | 53.3 | 54.2 | 54.5 | 54.2 | 54.2 | 54.1 | 54.2 | 54.4 | 54.4 | 53.2 | 52.7 | 66.1 | | | | | | | | | | | | | | | | | | | |
| NC027554.1 Chobar Gorge virus | 50.9 | 50.6 | 52.3 | 52.2 | 51.9 | 50.5 | 50.6 | 51.3 | 51.7 | 51.2 | 50.4 | 51.4 | 52.3 | 50.9 | 52.9 | 54.6 | | | | | | | | | | | | | | | | | | |
| HQ266582.1 Tribec virus | 49.4 | 48.6 | 49.2 | 50 | 47.1 | 47.7 | 47.9 | 48 | 48.4 | 48 | 48.3 | 48.2 | 49.4 | 48.6 | 50 | 50.8 | 55.2 | | | | | | | | | | | | | | | | | |
| KC288131.1 Kemerovo virus | 48.6 | 47.7 | 50.1 | 49.5 | 47.4 | 47.3 | 47.4 | 48.3 | 48.1 | 48.4 | 48.4 | 48.4 | 49.5 | 50.1 | 50.4 | 51 | 54.8 | 76.1 | | | | | | | | | | | | | | | | |
| KY023330.1 Okhotskiy virus | 48.8 | 47.4 | 49.9 | 49.5 | 48.4 | 46.9 | 47.5 | 48 | 48 | 48.1 | 48.7 | 48.4 | 49 | 50.3 | 50.7 | 51.6 | 54 | 72.3 | 72.2 | | | | | | | | | | | | | | | |
| NC014523.1 Great Island virus | 49.2 | 48.6 | 49.8 | 49.6 | 47.4 | 47.7 | 48 | 48.6 | 48.6 | 48.7 | 48.7 | 48.2 | 49.6 | 49.8 | 51.6 | 51.6 | 54.2 | 72.4 | 72.9 | 84.8 | | | | | | | | | | | | | | |
| NC027535.1 Chenuda virus | 48.9 | 49 | 50.3 | 50.1 | 49.3 | 48.2 | 48.1 | 49.5 | 49.8 | 49.8 | 50 | 49.8 | 50.2 | 48.8 | 51.6 | 51.5 | 53.8 | 61.3 | 61.1 | 60.7 | 60.6 | | | | | | | | | | | | | |
| NC027539.1 Wad Medani virus | 48.7 | 48.6 | 50.6 | 50.2 | 48.2 | 47.2 | 46.9 | 48.3 | 48.3 | 47.9 | 47.6 | 48.4 | 49.2 | 47.7 | 49.2 | 49.7 | 53 | 59.5 | 57.5 | 58.5 | 58.2 | 57.8 | | | | | | | | | | | | |
| MG770352.1 Kammavanpettai virus | 52 | 52.4 | 54.1 | 53.1 | 50.2 | 50.5 | 50.4 | 51.3 | 51.6 | 51.6 | 52 | 51.5 | 50.4 | 49.5 | 51.3 | 52.5 | 50.8 | 47.7 | 49.5 | 49 | 48.2 | 48.9 | 48.4 | | | | | | | | | | | |
| FJ969721.1 Bluetongue virus | 46.4 | 46.4 | 46.6 | 46.8 | 48 | 47.8 | 47.9 | 47.4 | 47.6 | 47.9 | 48.3 | 47.2 | 48.7 | 47.1 | 47.7 | 47.3 | 45.2 | 44.2 | 44.5 | 45.1 | 43.9 | 45.5 | 43.6 | 47.2 | | | | | | | | | | |
| NC013398.1 Epizootic hemorrhagic disease virus | 46.8 | 46.4 | 47.6 | 46.5 | 48.9 | 48.7 | 48.7 | 47.6 | 47.5 | 47.3 | 49.9 | 48.7 | 48.8 | 48 | 47.7 | 47.8 | 46.3 | 44.6 | 44.1 | 45.7 | 45.4 | 45.7 | 44.9 | 46.9 | 70.8 | | | | | | | | | |
| JQ070378.1 Eubenangee virus | 46.4 | 47.5 | 47.3 | 46.6 | 47.8 | 47.5 | 47.1 | 47.2 | 47.1 | 47.2 | 48.5 | 47.7 | 47.5 | 46.7 | 47.8 | 48.9 | 46.4 | 44.1 | 43.7 | 45.2 | 44.6 | 44.3 | 45.1 | 47.1 | 64.8 | 65.5 | | | | | | | | |
| NC022555.1 Wallal virus | 47.6 | 48 | 48.9 | 48.4 | 49.5 | 49.4 | 49.5 | 48.7 | 48.7 | 48.7 | 49.2 | 49.2 | 48.6 | 49.3 | 48.7 | 49.3 | 46.5 | 42.9 | 43 | 43.8 | 44.2 | 45.3 | 43.8 | 48.8 | 66.5 | 65.7 | 67.2 | | | | | | | |
| JQ610657.1 Changuinola virus | 47.6 | 47.9 | 47.2 | 47.1 | 48 | 47.5 | 47.7 | 48 | 47.9 | 47.8 | 48.2 | 48.4 | 48.7 | 47.2 | 48.4 | 48.4 | 45.7 | 44.1 | 44.7 | 45.1 | 44.7 | 45 | 44.1 | 47.9 | 63.7 | 65.3 | 64.4 | 66.6 | | | | | | |
| NC005989.1 Palyam virus | 47 | 46.3 | 46.9 | 46.9 | 48.1 | 47.7 | 48 | 48 | 48 | 47.7 | 49 | 48.3 | 48.2 | 47.4 | 47.8 | 48.3 | 44.3 | 43.2 | 43.4 | 43.4 | 44.1 | 44.5 | 43.4 | 48.1 | 58.8 | 61 | 59.9 | 62.7 | 61.8 | | | | | |
| JQ610666.1 Lebombo virus | 45.7 | 45.2 | 44.7 | 45.2 | 44.4 | 43.8 | 43.7 | 44.3 | 44.4 | 44.1 | 44.1 | 44.4 | 44 | 44.2 | 45.5 | 46.3 | 45.4 | 44.7 | 44.4 | 45.6 | 45.2 | 44.5 | 45.6 | 46.3 | 58.7 | 58.8 | 59.6 | 59.7 | 58.2 | 60.8 | | | | |
| JQ610677.1 Orungo virus | 45.5 | 45.8 | 46.2 | 45.2 | 46.2 | 46.8 | 47 | 45.2 | 45.2 | 45.6 | 46.5 | 46.2 | 46.2 | 46.6 | 45.7 | 47.6 | 44.5 | 44.1 | 44.4 | 44.2 | 45 | 44.8 | 44 | 46.5 | 57.2 | 58 | 57.6 | 59.1 | 57.2 | 60.2 | 62.6 | | | |
| HQ630904.1 Equine encephalosis virus | 44 | 45.6 | 46.1 | 45.7 | 45.6 | 45.4 | 45.3 | 45.1 | 44.9 | 45.1 | 45.9 | 45.5 | 44.3 | 45.4 | 44.8 | 45.5 | 45 | 42.6 | 42.8 | 43.6 | 43.3 | 43.6 | 43.7 | 44.9 | 55.9 | 55.3 | 55.6 | 54.7 | 54.6 | 55.8 | 57.5 | 57.2 | | |
| NC005998.1 St Croix River virus | 36.6 | 36.9 | 37.1 | 37.8 | 37.4 | 36.4 | 36.4 | 36.3 | 36.1 | 36.1 | 36.6 | 37.1 | 37.7 | 37.2 | 38.9 | 38.2 | 38.3 | 39.4 | 38.2 | 39.6 | 38.1 | 37.5 | 38.1 | 38.2 | 35.5 | 36.2 | 36.9 | 36.6 | 36.9 | 36.8 | 37.5 | 36 | 36 | |

**Figure S6.** Sequence identity matrix showing the nucleotide percentage similarity of novel orbiviruses to 28 other orbiviruses based on the VP3 (T2) gene.
